# Molecular, ecological, and behavioral drivers of the bat-virus relationship

**DOI:** 10.1016/j.isci.2022.104779

**Published:** 2022-07-20

**Authors:** Victoria Gonzalez, Arinjay Banerjee

**Affiliations:** 1 Vaccine and Infectious Disease Organization, University of Saskatchewan, Saskatoon, SK S7N 5E3, Canada; 2 Department of Veterinary Microbiology, University of Saskatchewan, Saskatoon, SK S7N 5B4, Canada; 3 Department of Biology, University of Waterloo, Waterloo, ON N2L 3G1, Canada; 4 Department of Laboratory Medicine and Pathobiology, University of Toronto, Toronto, ON M5S 1A8, Canada

**Keywords:** Virology, Evolutionary biology

## Abstract

Bats perform important ecological roles in our ecosystem. However, recent studies have demonstrated that bats are reservoirs of emerging viruses that have spilled over into humans and agricultural animals to cause severe diseases. These viruses include Hendra and Nipah paramyxoviruses, Ebola and Marburg filoviruses, and coronaviruses that are closely related to severe acute respiratory syndrome coronavirus (SARS-CoV), Middle East respiratory syndrome coronavirus (MERS-CoV), and the recently emerged SARS-CoV-2. Intriguingly, bats that are naturally or experimentally infected with these viruses do not show clinical signs of disease. Here we have reviewed ecological, behavioral, and molecular factors that may influence the ability of bats to harbor viruses. We have summarized known zoonotic potential of bat-borne viruses and stress on the need for further studies to better understand the evolutionary relationship between bats and their viruses, along with discovering the intrinsic and external factors that facilitate the successful spillover of viruses from bats.

## Graphical abstract

**Figure:**
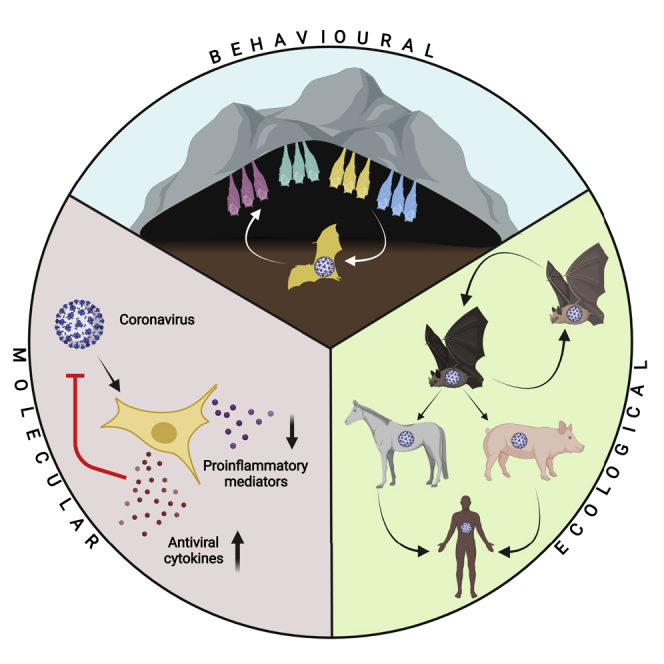

## Introduction

Bats belong to the order Chiroptera and are one of the most abundant and geographically diverse vertebrates in the world, representing over 20% of all mammals (Burgin et al., 2018). The order Chiroptera consists of more than 1400 species of bats and is further classified into two suborders, Yinpterochiroptera and Yangochiroperta, which diverged over 50 million years ago (Lei and Dong, 2016). These suborders represent biologically and ecologically diverse species that are globally distributed, sparing only the polar regions, extreme desert climates and few oceanic islands (Teeling et al., 2005; Nowak and Walker, 1994). Because of the essential ecological roles performed by bats, they have become keystone species of global ecosystems from which humans directly benefit. Bats help with controlling nocturnal insects and pests, thus benefiting humans and agriculture. Bats play a vital role in reseeding deforested lands, pollinating wild plants which provide food for humans and other species, and aid in the production of biological fertilizer through their guano (Kunz et al., 2011).

Despite performing critical ecological roles, bats are an emerging reservoir host for important viruses that cause significant pathology in humans and animals. Seventy percent of emerging and re-emerging infectious diseases are from an animal origin or zoonotic (Jones et al., 2008), and bats are known to host viruses that have played a role in zoonotic outbreaks. Some of these zoonotic agents include Ebola and Marburg filoviruses, Nipah and Hendra paramyxoviruses, and a diverse range of coronaviruses, including severe acute respiratory syndrome coronavirus (SARS-CoV), Middle East respiratory syndrome coronavirus (MERS-CoV), porcine epidemic diarrhea virus (PEDV) and swine acute diarrhea syndrome coronavirus (SADS-CoV) (Li et al., 2005; Memish et al., 2013; Huang et al., 2013; Schountz, 2014; Moratelli and Calisher, 2015). Most recently, a novel coronavirus, SARS-CoV-2, is speculated to have evolved in bats (Zhou et al., 2020). Despite evidence of bats harboring numerous viruses, naturally or experimentally infected bats do not demonstrate ostensible disease, with the exception of infection with rabies and related lyssaviruses, and Tacaribe virus (Davis et al., 2005; Cabrera-Romo et al., 2014; Paweska et al., 2016; Stockmaier et al., 2015; Mccoll et al., 2002; Cogswell-Hawkinson et al., 2012; Almeida et al., 2005). Multiple viruses that have spilled over from bats to humans, either directly or through an intermediate animal host, often cause severe and fatal disease in the spillover species.

While recent reviews have summarized the diversity of viruses detected in bats and underlying mechanisms that may contribute to viral tolerance in these flying mammals (Letko et al., 2020; Irving et al., 2021; Banerjee et al., 2020a), an updated and comprehensive review on the molecular, ecological, and behavioral features of bats that make them competent viral hosts does not exist. Here we have summarized major viral species that have been detected in bats, discussed the zoonotic potential of bat-borne viruses, and have highlighted knowledge gaps in our understanding of bats as reservoirs of emerging viruses.

### Potential factors that contribute to the viral reservoir status of bats

#### Flight and long-distance movement

**Figure 1:**
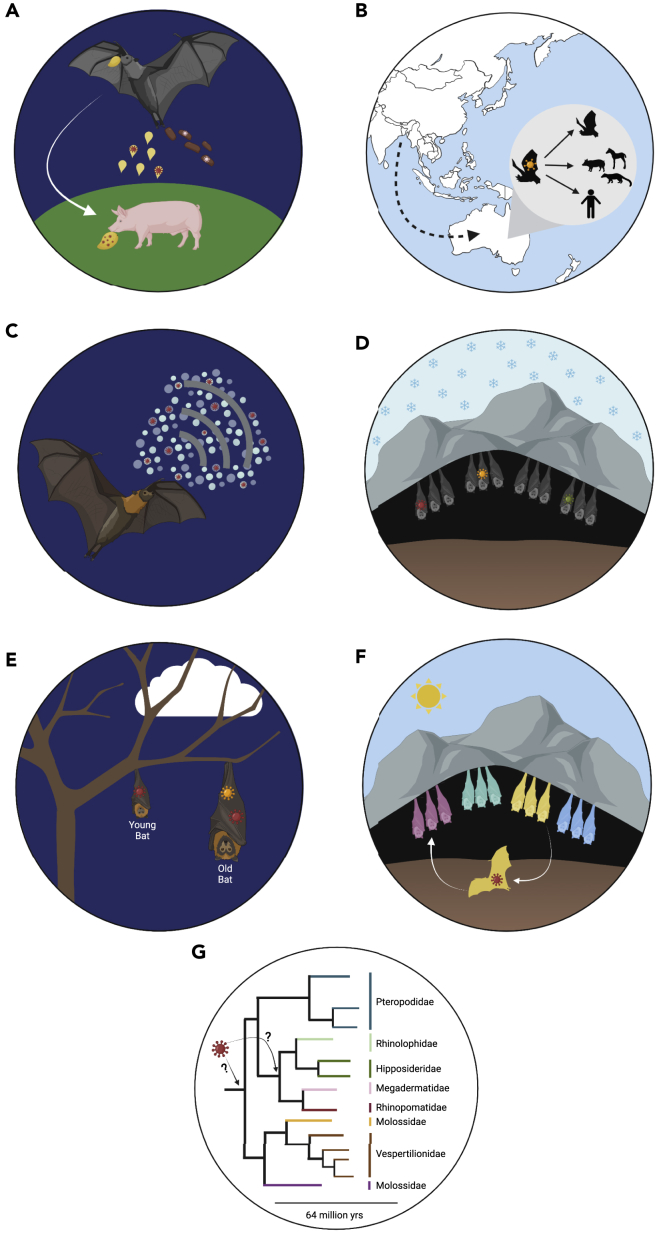
Ecological and behavioral aspects that influence the viral reservoir status of bats The order Chiroptera is composed of over 1400 bat species that span various ecological and behavioral traits. (A) For instance, bats are the only mammals capable of true flight. During flight, the core body temperature of bats, such as the little brown bat (*Myotis lucifugus*), rises to over 40°C (Hock, 1951). Some viruses that have been identified in bats, such as filoviruses, are capable of replicating in selected bat cells (*Hypsignathus monstrosus*, *Epomops buettikoferi*, *Rousettus aegyptiacus*, *Artibeus jamaicensis*, *Tadarida brasiliensis*, and *Eptesicus fuscus*) at temperatures attained during flight, suggesting that some viruses may have adapted to replicate or co-exist in their bat hosts even at elevated body temperatures (Miller et al., 2016). In addition, ebolavirus and bat paramyxoviruses have been detected in fecal matter and/or urine of experimentally and naturally infected bats (Swanepoel et al., 1996; Peel et al., 2019), suggesting that some viruses may be transmitted during defecation. Bats are also capable of discarding food remnants, such as fruits, which may be contaminated with virus-infected biological matter, allowing foraging animals to potentially become infected upon consumption (Chua et al., 2002; Nikolay et al., 2019). (B) The ability of bats to fly long distances may allow for the transmission of novel viruses and variants amongst bat populations and potentially into humans and other animals. (C) Yangochiropterans, like *Myotis lucifugus* (Kazial et al., 2008), use laryngeal echolocation to produce sounds from the mouth or nose (Neuweiler, 2000). Therefore, it might be possible for these bat species to produce aerosols while echolocating, which would potentially allow for the dissemination of viruses that replicate within the respiratory tract, such as rabies, Aravan, Khujand, and Irkut viruses (Constantine et al., 1972; Hughes et al., 2006). The relation between echolocation and aerosol production in bats remains to be mechanistically investigated. (D) Temperate bats are known to hibernate during winter, allowing viruses, such as rabies virus, to be maintained for extended periods of time (George et al., 2011). The role of hibernation and daily torpor in facilitating virus tolerance and persistence in bats remains understudied. (E) Hibernation, amongst other factors, positively influences the exceptional lifespan of bats (Wilkinson and South, 2002). The immunological consequences of aging and associated control over virus replication and persistence remain unknown for bats. (F) Bats are gregarious species and roost in multi-species colonies. Living in dense clusters may facilitate the spread of viruses and other pathogens between different bat species and within immune and naive individuals of the same species, as described for European bat lyssavirus (López-Roig et al., 2014). (G) As the order Chiroptera diverged over 64 million years ago, bats may have co-evolved with some viral families to develop fine-tuned antiviral responses that limit host damage and promote viral tolerance. The phylogenetic tree was adapted from the phylogenomic analyses performed by Lei and Dong (Lei and Dong, 2016). Created with BioRender.com.

Powered flight has evolved independently in four animal groups: pterosaurs, insects, birds, and bats, with chiropterans being the only mammals capable of flight (Figure 1A). It has been postulated that the evolution of flight may allow bats to control viral infections (O'Shea et al., 2014). During flight, the body temperature of the little brown bat (*Myotis lucifugus*) rises to over 40°C, a temperature that is hypothesized to be less optimal for pathogen survival and is representative of a fever response in humans (Hock, 1951; O’Grady et al., 1998). Thus, this elevation in temperature during flight may aid in controlling infection or infection spread while accelerating immune processes (Kluger et al., 1998), as posited for bats by O’Shea et al. (O'Shea et al., 2014). However, viruses such as Marburg, Angola, and Ebola filoviruses are capable of replicating in six bat cell lines when incubated at temperatures attained during flight. These cell lines were derived from the hammer-headed fruit bat (*Hypsignathus monstrosus*), Buettikofer’s epauletted fruit bat (*Epomops buettikoferi*), the Egyptian fruit bat (*Rousettus aegyptiacus*), the Jamaican fruit bat (*Artibeus jamaicensis*), the Mexican free-tailed bat (*Tadarida brasiliensis*), and the big brown bat (*Eptesicus fuscus*), with virus propagation occurring when incubated at temperatures ranging from 37°C to 41°C (Miller et al., 2016). In addition, ebolavirus and multiple bat paramyxoviruses have been detected in the fecal matter and/or urine of experimentally and naturally infected fruit bats, such as the Wahlberg’s epauletted fruit bat (*Epomorphorus wahlbergi*) and flying foxes (*Pteropus* spp*.*) (Swanepoel et al., 1996; Peel et al., 2019), suggesting that bats may shed viruses through feces (Figure 1A). Despite these studies, the role of flight and fever in shaping the bat antiviral immune response remains to be mechanistically studied. Flight alone is unlikely to put sufficient evolutionary pressure on pathogens to adapt to cellular responses, such as interferons, that are not induced during flight (Schountz et al., 2017). Moreover, currently reported flight temperatures in bats may be an overestimation of temperatures attained during flight because of limited methodology (Levesque et al., 2021). Even so, currently reported body temperatures in bats during flight are only high relative to humans and not other mammals, such as the Arabian oryx (*Oryx leucoryx*), southern flying squirrel (*Glaucomys volans*), and large treeshrews (*Tupaia tana*) that display an elevated body temperature of approximately 40°C (Levesque et al., 2018, 2021; Hetem et al., 2010).

The adaptation of flight also allows bat species to undergo significant movements upon seasonal changes (Figure 1B). Although seasonal migration is much less common in tropical bats, some temperate species are known to fly extreme distances between seasonal roosts (Fleming, 2019; Griflin, 2012). For instance, *T. brasiliensis* are known to travel over 800 miles between their summer and overwintering sites (Russell et al., 2005). These large-scale migration events may allow for the transmission of novel viruses or variants into native bat populations, as bats from different species are known to roost together, although this remains to be scientifically demonstrated (Kelm et al., 2021). Although migration as a trait is unlikely to introduce new pathogens, previous work on migratory songbirds suggest that migration may be associated with potential immunosuppression and resurgence of chronic viral infections (Becker et al., 2020a; Plowright et al., 2016).

#### Echolocation

In addition to flight, bats are one of two land mammals that have evolved to use echolocation to position themselves to their immediate physical surroundings in the dark and locate prey (Jones and Teeling, 2006). Several forms of echolocation exist in chiropterans; however, there are two overarching forms: laryngeal and tongue-clicking. Not all bats can utilize echolocation for navigation, such as bats in the family Pteropodidae. However, one genus in the Pteropodidae family (*Rousettus* spp.) has re-evolved to utilize tongue clicking to navigate (Holland et al., 2004). A potential outcome of echolocation is the aerosolization of the oropharyngeal fluids, mucus, or saliva, allowing for the dissemination of viruses that are capable of replicating in the respiratory tract or oral cavity (Figure 1C), such as rabies, Aravan, Khujand, and Irkut viruses (Constantine et al., 1972; Hughes et al., 2006). However, the role of echolocation in facilitating pathogen spread remains to be experimentally tested.

#### Torpor and hibernation

Variation in weather, both daily and seasonally, may have considerable energetic costs, forcing endotherms, such as bats, to find ways to conserve energy to maintain a stable body temperature. To compensate for lost energy, animals may increase their foraging rates; however, many available food sources vary by weather conditions and seasons. Both torpor and hibernation are widespread strategies employed by temperate bats allowing them to reduce their energy requirements and thus, saving their energy for when foraging opportunities are energetically less costly to pursue (Reeder and Cowles, 1951). This is important as some temperate bats are insectivorous and insect availability also varies by season and time of day (Stawski, 2012), causing insectivorous bats to reduce their activity levels appropriately. Tropical and sub-tropical bats, such as the Peter’s tent-making bat (*Uroderma bilobatum*), have been identified to utilize a novel, cyclic, bradycardic state to reduce their daily expenditure of energy instead of torpor as the ambient temperature is too high for bats to sufficiently lower their body temperature (O'Mara et al., 2017). In addition to foraging, flight, echolocation, and thermoregulation are all energetically demanding processes (Currie et al., 2015; Lyman, 1970; Winter and Von Helversen, 1998).

Though hibernation is beneficial for the longevity of bats, it also enables the overwintering of viruses (Figure 1D). George et al. have demonstrated that viruses that have long incubation periods, such as rabies virus, provide infected bats with enough time to enter hibernation, allowing the cold temperature to suppress viral activity (George et al., 2011). This suppression makes hibernating bats a temporal maintenance reservoir, enabling the preservation of the virus until birthing season, where a surplus of naive bats is available. This permits the avoidance of epizootic fadeout. Similar findings were also noted for coronaviruses, where researchers have demonstrated the overwintering of various alphacoronaviruses in hibernating *M. lucifugus* and the sharing of these viruses between different bat species at co-hibernation sites (Subudhi et al., 2017; Misra et al., 2009; Dominguez et al., 2007). In addition, the reduction in body temperature during daily torpor has been speculated to be a strategy to resist viral infection (LeGrand and Alcock, 2012). However, this strategy may have also led to molecular co-adaptations in the viruses themselves, which may favor co-existence of these viruses alongside their bat hosts. Indeed, bats and naturally existing viruses provide an intriguing model to study virus-host co-evolution.

#### Longevity

For most mammalian species, the larger their size, the longer they live; however, some bat species have longer lifespans despite their small body size (Austad and Fischer, 1991). Intriguingly, some females that are larger than males have a shorter lifespan (Podlutsky et al., 2005). Moreover, males of some harem polygynous bat species are larger than the females, but have a shorter lifespan (Wilkinson et al., 2016). The lifespan of bats has been documented to be over 20 years for 22 bat species, more than 30 years for six species, and a phenomenal 41 years for one species (*Myotis brandtii*) (Wilkinson and South, 2002; Podlutsky et al., 2005). Furthermore, a recent study by Austad et al. reported that only 19 species of mammals are longer-lived than humans when adjusted for body size, and 18 out of 19 species are bats (Austad, 2010).

There are several features of bats that favor their long lifespan, such as their low reproductive rate (Barclay et al., 2003; Speakman, 2008), the escape of predators by flight (Holmes and Austad, 1994), cooperative social behavior (Wilkinson and Adams, 2019), body mass (Wilkinson and Adams, 2019), cave use (Wilkinson and Adams, 2019), and their ability to undergo daily torpor and seasonal hibernation (Podlutsky et al., 2005; Turbill et al., 2011), with hibernating species living approximately six years longer than non-hibernating species (Wilkinson and South, 2002). In addition, recent studies have now shed light on molecular processes that perhaps enable some bat species to live a long life, such as the shortening of telomeres with age that was observed in the greater horseshoe bat (*Rhinolophus ferrumequinum*) and the common bent-wing bat (*Miniopterus schreibersii*), but not in the free-living greater mouse-eared bats (*Myotis myotis*) (Foley et al., 2018). Wilkinson and Adams have further demonstrated that longevity has evolved in bats at least four separate times. These include horseshoe bats (*Rhinolophus* spp.), the common vampire bat (*Desmodus rotundus*), long-eared bats (*Plecotus* spp.), and at least one *Myotis* lineage (Wilkinson and Adams, 2019).

In addition to an expanded lifespan, bats possess features that favor virus tolerance. For instance, adaptations in genes that maintain genome integrity have been reported for bats. Huang et al. have demonstrated advantageous positive selection in cellular enzymes that play a vital role in DNA repair and damage signaling pathways in *Myotis myotis* (Huang et al., 2016, 2019). These adaptations are consistent with the low levels of cancer observed in bats (Seluanov et al., 2018). Moreover, numerous double-stranded DNA break repair genes are under positive selection in at least two bat species, the black flying fox (*Pteropus alecto*) and David’s myotis (*Myotis davidii*), suggesting the ability of these bats to resist DNA damage (Zhang et al., 2013). The double-stranded DNA repair pathway is also strongly correlated with longevity (Tian et al., 2019). Gorbunova et al. have recently reported evidence of mutations in the growth hormone receptor and insulin-like growth factor 1 receptor (Seim et al., 2013), higher levels of antioxidant activity across tissues (Conde-Pérezprina et al., 2012), and enhanced autophagy activity with advanced aging in long-lived bat species (Huang et al., 2019), such as the Brandt’s bat (*Myotis brandtii*), *Desmodus rotundus*, and *Myotis myotis* (Gorbunova et al., 2020). Overall, the parameters that enable the long lifespan of bats may be a double-edged sword, which may protect bats from severe viral disease, but may also facilitate the long-term maintenance of certain viruses in their long-lived hosts (Figure 1E).

#### Roosting behavior

Numerous structures are known to support bat roosts, including caves, tree foliage, tree cavities, rock crevices, and man-made structures (Kunz, 1982). Because there are over 1400 bat species, many overlap in their global distribution with numerous regions being home to over 40 species (Luis et al., 2015). Because many chiropterans are gregarious, living in dense clusters and in certain roosting sites can facilitate intra- and inter-species transmission of pathogens and sustain acute infections (Figure 1F). Willoughby et al. have reported that cave-roosting behavior is an important driver of virus sharing in bats, with caves housing the largest aggregates of bats in the world (Willoughby et al., 2017). These co-roosting sites can be composed of bat species that would not typically interact while outside of the roost (Willoughby et al., 2017), promoting the circulation and maintenance of viruses in different bat species in addition to facilitating virus host switch. Roost size and species richness have also been demonstrated to be positively linked to the dynamics of European bat lyssavirus 1, where Lopez-Roig et al. have detected neutralizing antibodies in nine species within a multi-species bat colony (López-Roig et al., 2014).

#### Feeding habits

Because of foraging by flight, chiropterans may be constrained by the amount of food that they can ingest. While feeding on fruit or insects, partially digested food can be discarded on the ground. These discarded remnants may be contaminated with viruses from residual bat saliva and therefore, pose a risk of infection for other foraging mammals (Figure 1A). This sequence of events has been linked to viral outbreaks, including the Nipah virus outbreaks in Malaysia and Bangladesh (Nikolay et al., 2019; Chua et al., 2002) and the Hendra virus outbreak in Queensland, Australia (Selvey et al., 1995). In both cases, the consumption of food contaminated with urine or saliva from infected pteropid bats was the likely source of virus introduction (Nikolay et al., 2019; Chua et al., 2002; Halpin et al., 2011). Factors that may affect the viability of microbes as they transition through the bat gut remain to be identified (Banerjee et al., 2018b). However, it must be noted that zoonotic transmission events are extremely transient and not all bats are infected or shedding virus constantly. It is also important to note that not all bat species are infected with similar viruses. Thus, it is critical that we do not extrapolate data from a handful of bat species to all known bat species.

#### Co-evolution with viruses and other known microbes

The interaction of a virus with its host involves a balance of actions and counteractions between the immune escape mechanisms of the pathogen and the immune system of the host. A tight genetic interaction between a pathogen and host can lead to ongoing host-pathogen co-evolution, in which adaptations and counter-adaptations occur. The origin of bats is estimated around 64 million years ago (Teeling et al., 2005), providing ample time for the co-evolution processes between bats and their viruses to occur (Figure 1G). Overall, hundreds of viruses have been identified in bats, including those which consist of an RNA (Table 1) or DNA (Table 2) genome. Some bat species also demonstrate co-evolutionary associations with other microbes, such as *Trypanosoma cruzi* (Hamilton et al., 2012), *Plasmodium* spp*.* (Schaer et al., 2013), *Bartonella* spp. (Kosoy et al., 2010), *Mycoplasma* spp*.* (Becker et al., 2020b), and herpesviruses (Griffiths et al., 2020). It is difficult to study co-evolution of viruses and bats in real-time, but ongoing research has identified multiple adaptations that suggest that the bat immune system may have evolved overtime to better tolerate virus infection and minimize disease severity (Banerjee et al., 2020a; Irving et al., 2021).

**Table 1:** An updated list of RNA viruses detected in bats

Viral Family	Genera	Viral species	Bat species	Refs
Arenaviridae	*Mammarenavirus*	Tacaribe virus	*Artibeus jamaicensis, Artibeus lituratus, Desmodus rotundus, Platyrrhinus helleri, Tyloncteris robustula*	(Calisher et al., 2006; Beltz, 2017)
Astroviridae	Mamastrovirus	Mamastrovirus spp.	*Eonycteris spelaea, Hipposideros pomona, Ia io, Megaderma lyra, Myotis horsfieldii, Pipistrellus kuhlii, Rhinolophus affinis, Rhinolophus lepidus, Rousettus amplexicaudatus, Rousettus leschenaultii, Scotophilus kuhlii*	(Chen et al., 2014)
	Unclassified	Astrovirus spp.	*Artibeus planirostris, Eidolon helvum, Hipposideros caffer, Hipposideros cineraceus, Miniopterus griveaudi, Miniopterus mossambicus, Miniopterus schreibersii, Mops condylurus, Mormopterus francoismoutoui, Myotis daubentonii, Myotis goudoti, Myotis nattereri, Nyctalus noctule, Nycteris thebaica, Pipistrellus pygmaeus, Plecotus auritus, Pteropus giganteus, Rousettus madagascariensis, Triaenops afer, Triaenops furculus, Triaenops menamena*	(Chen et al., 2014)
		Bat astrovirus	*Barbastella barbastellus, Coleura afra, Desmodus rotundus, Eptesicus andersoni, Eptesicus serotinus, Hipposideros armiger, Hipposideros caffer, Hipposideros gigas, Hipposideros larvatus, Hipposideros lekaguli, Hipposideros pomona, Hipposideros turpis, Hypsugo savii, Ia io, Megaderma lyra, Megaerops kusnotoi, Miniopterus fuliginosus, Miniopterus inflatus, Miniopterus magnater, Miniopterus pusillus, Miniopterus schreibersii, Myotis bechsteinii, Myotis capaccinii, Myotis chinensis, Myotis daubentonii, Myotis marodactylus, Myotis myotis, Myotis blythii, Myotis mystacinus, Myotis nattereri, Myotis pequinius, Myotis petax, Myotis ricketti, Mytois emarginatus, Mytois fimbriatus, Mytois horsfieldii, Nyctalus noctula, Pipistrellus pipistrellus, Pipistrellis abramus, Pipistrellus kuhlii, Plecotus auritus, Rhinolophus affinis, Rhinolophus ferrumequinum, Rhinolophus hipposideros, Rhinolophus pearsonii, Rhinolophus rouxi, Rhinolophus rouxii, Rhinolophus thomasi, Rhinopoma hardwickii, Rousettus aegyptiacus, Rousettus leschenaultii, Scotophilus kuhlii, Tadarida brasiliensis, Taphozous melanopogon, Taphozous perforates, Tyloncyteris robustula, Vesperitilio murinus*	(Beltz, 2017; Chen et al., 2014)
Bornaviridae	Unclassified	Bat bornavirus	*Myotis nattereri, Pipistrellus pipstrellus*	(Chen et al., 2014)
Caliciviridae	*Norovirus*	Bat norovirus	*Rhinolophus affinis, Rhinolophus sinicus*	(Chen et al., 2014)
	*Sapovirus*	Bat sapovirus	*Eidolon helvum, Hipposideros pomona*	(Chen et al., 2014; Beltz, 2017)
	Unclassified	Calicivirus spp.	*Mystacina tuberculata, Myotis alcathoe*	(Chen et al., 2014)
		Bat calicivirus	*Eptesicus serotinus, Mops condylurus, Myotis daubentonii, Myotis myotis, Pipistrellus subflavus, Rhinolophus ferrumequinum, Rhinolophus sinicus, Rousettus aegyptiacus*	(Chen et al., 2014)
Coronaviridae	*Alphacoronavirus*	Bat alphacoronavirus spp.	*Afronycteris nana, Aselliscus stoliczkanus, Chalinolobus gouldii, Chalinolobus morio, Cloeotis percivali, Desmodus rotundus, Eptesicus regulus, Eptesicus vulturnus, Falsistrellus mackenziei, Hipposideros* spp*., Hypsugo* spp*., Miniopterus fuliginosus, Miniopterus fuscus, Miniopterus natalensis, Miniopterus schreibersii, Mops pumilus, Myotis brandtii, Myotis chinensis, Myotis daubentonii, Myotis myotis, Myotis ricketti, Neoromicia capensis, Neoromicia nanus, Nyctalus lasiopterus, Nycteris* spp*., Pipistrellus abramus, Rhinolophus* spp*., Rhinopoma hardwickii, Scotophilus dinganii, Tadarida brasiliensis, Tylonycteris robustula*	(Chen et al., 2014)
		Alphacoronavirus spp.	*Artibeus planirostris, Carollia perspicillata, Chaerephon plicatus, Eptesicus isabellinus, Hipposideros cineraceus, Hipposideros larvatus, Hipposideros pomona, Miniopterus natalensis, Miniopterus schreibersii, Murina cyclotis, Myotis capaccinii, Myotis daubentonii, Myotis emarginatus, Myotis horsfieldii, Myotis laniger, Myotis muricola, Myotis myotis, Myotis nattereri, Myotis punicus, Neoromicia capensis, Nycteris thebaica, Pipistrellus khulii, Pipistrellus pipistrellus, Rhinolophus ferrumequinum, Rhinolophus malayanus, Rhinolophus simulator, Rhinolophus sinicus, Rhinolophus stheno, Scotophilus kuhlii*	(Chen et al., 2014)
		HCoV-229E	*Hipposideros caffer, Hipposideros curtus, Hipposideros ruber, Hipposideros vittatus, Pipistrellus inexspectatus*	(Chen et al., 2014)
		229E-related bat CoV	*Hipposideros abae, Hipposideros caffer, Hipposideros ruber, Hipposideros vittatus, Rousettus aegyptiacus*	(Chen et al., 2014)
		HCoV-NL63	*Triaenops afer.*	(Chen et al., 2014)
		Porcine epidemic diarrhea virus	*Myotis horsfieldii*	(Chen et al., 2014)
	*Betacoronavirus*	Bat betacoronavirus spp.	*Carollia perspicillata, Cynopterus brachyotis, Cynopterus sphinx, Eidolon helvum, Eoncyteris spelaea, Eptesicus nilssoni, Hipposideros armiger, Hipposideros pomona, Hipposideros pratti, Hypsugo pulveratus, Ia io, Macroglossus minimus, Myotis daubentonii, Myotis emarginatus, Myotis horsfieldii, Neoromicia capensis, Pipistrellus abramus, Pipistrellus hesperidus, Rhinolophus affinis, Rhinolophus blasii, Rhinolophus euryale, Rhinolophus ferrumequinum, Rhinolophus hipposideros, Rousettus leschenaultii, Tylonycteris pachypus, Vespertilio sinensis*	(Chen et al., 2014)
		Betacoronavirus spp.	*Eptesicus isabellinus, Eptesicus serotinus, Hypsugo savii, Micropteropus pusillus, Miniopterus natalensis, Myotis emarginatus, Nyctalus noctula, Nycteris thebaica, Pipistrellus kuhlii, Pteronotus parnellii, Pteropus alecto, Rhinolophus ferrumequinum, Rhinolophus hipposideros, Rhinolophus malayanus, Rhinolophus pusillus, Rhinolophus sinicus, Rhinolophus stheno*	(Chen et al., 2014)
		SARS-CoV	*Rhinolophus* spp	(Chen et al., 2014; Calisher et al., 2006; Ravelomanantsoa et al., 2020)
		Bat SARS-like CoV	*Aselliscus stoliczkanus, Hipposideros armiger, Hipposideros pomona, Hipposideros pratti, Miniopterus schreibersii, Rhinolophus ferrumequinum, Rhinolophus hipposideros, Rhinolophus monoceros, Rhinolophus pearsonii, Rhinolophus pusillus, Rhinolophus rex, Rhinolophus sinicus, Rhinolophus thomasi*	(Chen et al., 2014)
		SARS-CoV-2	*Rhinolophus* spp.	(Ravelomanantsoa et al., 2020)
		Bat MERS-like CoV	*Hypsugo pulvertaus, Ia io, Vepertilio sinensis*	(Chen et al., 2014; Ravelomanantsoa et al., 2020)
	Unclassified	Bat coronavirus	*Numerous bat species, essentially worldwide.*	(Chen et al., 2014)
		Swine acute diarrhea syndrome related coronavirus	*Hipposideros* spp., *Myotis* spp*., Pipistrellus* spp*., Rhinolophus* spp*.*	(Chen et al., 2014)
Filoviridae	*Ebolavirus*	Bombali virus	*Chaerephon pumilus, Mops condylurus, Mops pumilus*	(Chen et al., 2014; Markotter et al., 2020; Goldstein et al., 2018)
		Reston ebolavirus	*Acerodon jubatus, Chaerephon plicatus, Cynopterus brachyotis, Cynopterus sphinx, Hipposideros pomona, Miniopterus australis, Miniopterus schreibersii, Myotis ricketti, Pipistrellus pipistrellus, Pteropus vamprus, Rhinolophus affinis, Rousettus amplexicaudatus, Rousettus leschenaultii, Scotophilus kuhlii*	(Beltz, 2017; Olival and Hayman, 2014)
		Zaire ebolavirus	*Cynopterus sphinx, Eidolon helvum, Epomops franqueti, Epomophorus gambianus, Hipposideros pomona, Hypsignathus monstrosus, Micropteropus pusillus, Miniopterus schreibersii, Mops condylurus, Myonycteris torquata, Myotis* spp*., Pipistrellus pipistrellus, Rousettus aegyptiacus, Rousettus leschenaulti*	(Beltz, 2017; Olival and Hayman, 2014; Markotter et al., 2020)
	*Cuevavirus*	Lloviu virus	*Miniopterus schreibersii*	(Beltz, 2017; Olival and Hayman, 2014)
	*Dianlovirus*	Mengla virus	*Rousettus* spp*.*	(Chen et al., 2014; Yang et al., 2019)
	*Marburgvirus*	Marburg virus	*Epomops buettikoferi, Epomops franqueti, Hipposideros* spp*., Hypsignathus monstrosus, Micropteropus pusillus, Miniopterus inflatus, Rhinolophus eloquens, Rousettus aegyptiacus*	(Beltz, 2017; Olival and Hayman, 2014)
		Ravn virus	*Rousettus aegyptiacus*	(Jones et al., 2019)
	Unclassified	Bat filovirus	*Eonycteris spelaea, Rousettus* spp*.*	(Chen et al., 2014)
Flaviviridae	*Flavivirus*	Banzi virus	*Eidolon helvum, Epomophorus anuras, Miniopterus schreibersii, Mops condylurus, Tadarida pumila*	(Fagre and Kading, 2019)
		Bat flavivirus	*Epomops franqueti, Hipposideros gigas, Rousettus aegyptiacus*	(Chen et al., 2014)
		Bussuquara virus	*Artibeus jamaicensis*	(Fagre and Kading, 2019)
		Bukalasa bat virus	*Chaerephon pumilus, Mops condylurus, Tadarida pumila*	(Calisher et al., 2006; Beltz, 2017; Kading and Schountz, 2016)
		Carey Island virus	*Cynopterus brachyotis, Macroglossus minimus*	(Calisher et al., 2006; Kading and Schountz, 2016)
		Central European encephalitis virus	Unidentified bat	(Fagre and Kading, 2019)
		Dakar bat virus	*Chaerephon pumilus, Mops condylurus, Scotophilus* spp*., Taphozous perforatus*	(Calisher et al., 2006; Beltz, 2017; Kading and Schountz, 2016)
		Dengue virus	*Anoura geoffroyi, Artebius cinereus, Artibeus intermedius, Artibeus jamaicensis, Artibeus lituratus, Artibeus planirostris, Carollia brevicauda, Carollia perspicillata, Desmodus rotundus, Eumops glaucinus, Glossophaga soricina, Molossus ater, Molossus molossus, Molossus pretiosus, Molossus rufus, Molossus sinaloae, Myotis lucifugus, Myotis nigricans, Natalus stramineus, Phyllostomus discolor, Phyllostomus hastatus, Pteronotus davyi, Pteronotus parnellii, Rhogeessa bickhami, Rhogeessa tumida*	(Beltz, 2017; Fagre and Kading, 2019; Chen et al., 2014)
		Entebbe bat virus	*Chaerephon pumilus, Mops condylurus, Tadarida limbata,*	(Calisher et al., 2006; Beltz, 2017; Kading and Schountz, 2016)
		Ilheus virus	*Anoura geoffroyi, Artebius cinereus, Artebius jamaicensis, Artibeus lituratus, Carollia perspicillata, Desmodus rotundus, Glossophaga soricina, Molossus ater, Molossus molossus, Natalus tumidirostris, Phyllostomus hastatus, Pteronotus davyi, Pteronotus parnellii, Sturnira* spp*., Vampyrops helleri*	(Beltz, 2017; Fagre and Kading, 2019)
		Israel turkey meningoencephalitis virus	*Rousettus aegyptiacus*	(Fagre and Kading, 2019)
		Japanese encephalitis virus	*Cynopterus brachyotis, Cynopterus sphynx, Eptesicus fuscus, Hipposideros armiger, Hipposideros terasensis, Hipposideros bicolor, Hipposideros cineraceus, Hipposideros pomona, Hipposideros speoris, Megaderma lyra, Miniopterus fuliginosus, Miniopterus shreibersii, Murina aurata, Murina leucogaster, Murina hilgendorfi, Myotis lucifugus, Myotis macrodactylus, Myotis mystacinus, Pipistrellus abramus, Pipistrellus subflavus, Pteronotus auritus, Pteropus alecto, Pteropus giganteus, Pteropus goldii, Pteropus scapulatus, Rhinolophus comutus, Rhinolophus ferrumequinum, Rhinolophus macrotus, Rhinolophus rouxi, Rousettus leschenaultii, Tadarida brasiliensis, Taphozous melanopogon, Vespertilio superans*	(Calisher et al., 2006; Beltz, 2017; Fagre and Kading, 2019; Kading and Schountz, 2016)
		Jugra virus	*Cynopterus brachyotis*	(Calisher et al., 2006; Kading and Schountz, 2016)
		Kyasanur forest disease virus	*Cynopterus sphinx, Pteropus giganteus, Rhinolophus rouxi, Rousettus leschenaultii*	(Calisher et al., 2006; Beltz, 2017; Fagre and Kading, 2019; Kading and Schountz, 2016)
		Montana Myotis leukoencephalitis virus	*Myotis lucifugus*	(Calisher et al., 2006; Kading and Schountz, 2016)
		Murray Valley encephalitis virus	*Eptesicus pumilus, Pteropus* spp*., Pteropus gouldi, Pteropus scapulatus*	(Fagre and Kading, 2019)
		Ntaya virus	*Eidolon helvum, Rousettus* spp*.*	(Fagre and Kading, 2019)
		Phnom-Penh bat virus	*Cynopterus brachyotis, Eonycteris spelaea*	(Calisher et al., 2006; Kading and Schountz, 2016)
		Rio Bravo virus	*Eptesicus fuscus, Molossus ater, Noctilio leporinus, Pteronotus parnellii, Tadarida brasiliensis, Tadarida mexicana*	(Calisher et al., 2006; Beltz, 2017; Kading and Schountz, 2016)
		St. Louis encephalitis virus	*Anoura geoffroyi, Artibeus intermedius, Artibeus jamaicensis, Artibeus litratus, Artibeus phaeotis, Carollia perspicillata, Eptesicus fuscus, Glossophaga soricina, Molossus ater, Molossus major, Molossus molossus, Myotis lucifugus, Natalus tumidirostris, Phyllostomus hastatus, Pteronotus davyi, Pteronotus parnellii, Sturnira lilium, Tadarida brasiliensis*	(Beltz, 2017; Fagre and Kading, 2019; Kading and Schountz, 2016)
		Saboya virus	*Nycteris gambiensis*	(Calisher et al., 2006)
		Sokoluk virus	*Pipistrellus pipistrellus*	(Calisher et al., 2006; Beltz, 2017; Kading and Schountz, 2016)
		Tamana bat virus	*Artebius* spp*., Carollia perspicillata, Desmodus rotundus, Glossophaga soricina, Molossus ater, Molossus major, Phyllostomus discolor, Phyllostomus hastatus, Pteronotus parnellii, Tadarida brasiliensis*	(Calisher et al., 2006; Beltz, 2017; Kading and Schountz, 2016)
		Tick-borne encephalitis virus	*Barbastella barbastellus, Myotis myotis, Plecotus auratus, Rhinolophus hipposideros*	(Fagre and Kading, 2019)
		Uganda S virus	*Rousettus* spp*., Tadarida* spp*.*	(Calisher et al., 2006; Kading and Schountz, 2016)
		Usutu virus	*Eidolon helvum, Rousettus aegyptiacus*	(Fagre and Kading, 2019)
		West Nile virus	*Artibeus jamaicensis, Artibeus lituratus, Eidolon helvum, Epomophorous minor, Eptesicus fuscus, Glossophaga soricina, Mops condylurus, Myotis lucifugas, Myotis lucifugus, Myotis nigricans, Myotis septentrionalis, Pteropus scapulatus, Rousettus aegyptiacus, Rousettus leschenaultii, Tadarida brasiliensis, Tadarida pumila*	(Beltz, 2017; Fagre and Kading, 2019)
		Yellow fever virus	*Artebius cinereus, Artibeus jamaicensus, Artibeus lituratus, Carollia perspicillata, Eidolon helvum, Epomophorus* spp*., Eptesicus fuscus, Glossophaga soricina, Molossus ater, Molossus molossus, Mops condylurus, Myotis lucifugus, Phyllostomus hastatus, Pteronotus davyi, Pteronotus davyi, Pteronotus parnellii, Rousettus aegyptiacus, Tadarida pumila, Vampyrops helleri*	(Beltz, 2017; Fagre and Kading, 2019)
		Yokose virus	*Myotis daubentonii, Miniopterus fuliginosus, Taphozous melanopogon*	(Beltz, 2017; Chen et al., 2014; Kading and Schountz, 2016)
		Zika virus	*Artibeus planirostris, Eidolon helvum, Mops condylurus, Rousettus aegyptiacus, Rousettus angolensis, Tadarida pumila*	(Fagre and Kading, 2019; Chen et al., 2014)
	*Hepacivirus*	Bat hepacivirus	*Glauconycteris* spp*., Hipposideros vittatus, Otomops martiensseni, Peropteryx macrotis, Scotoecus* spp*.*	(Chen et al., 2014)
		Hepacivirus spp.	*Desmodus rotundus*	(Chen et al., 2014)
	*Pegivirus*	Bat pegivirus	*Artibeus glaucus, Carollia perspicillata, Chaerephon* spp*., Coleura afra, Desomodus rotundus, Eidolon helvum, Epomophorus labiatus, Glossophaga commissarisi, Hipposideros gigas, Hipposideros vittatus, Megaloglossus woermanni, Mops condylurus, Nyctinomops macrotis, Otomops martiensseni, Pteropus giganteus, Rousettus aegyptiacus, Scotophilus dinganii, Sturnira lilium, Sturnira ludovici, Taphozous* spp*., Trachops cirrhosus*	(Chen et al., 2014)
	*Pestivirus*	Bat pestivirus	*Rhinolophus affinis, Scotophilus kuhlii*	(Beltz, 2017; Chen et al., 2014)
	Unclassified	Bat GB virus	*Rousettus aegyptiacus*	(Chen et al., 2014)
		Bat GB-like virus	*Eidolon helvum, Hipposideros gigas, Rousettus aegyptiacus*	(Chen et al., 2014)
Hantaviridae	*Loanvirus*	Brno virus	*Nyctalus noctula*	(Chen et al., 2014)
		Longquan virus	*Rhinolophus affinis, Rhinolophus monoceros, Rhinolophus sinicus*	(Beltz, 2017)
	*Mobatvirus*	Laibin virus	*Taphozous melanopogon*	(Beltz, 2017)
		Quezon virus	*Rousettus amplexicaudatus*	(Chen et al., 2014)
		Xuan Son virus	*Hipposideros cineraceus, Hipposideros pomona*	(Beltz, 2017; Chen et al., 2014)
	*Orthohantavirus*	Araraquara virus	*Anoura caudifer, Diphylla ecaudata*	(Beltz, 2017)
		Andes virus	*Carollia perspicillata, Desmodus rotundus*	(Chen et al., 2014)
		Dakrong virus	*Aselliscus stoliczkanus*	(Chen et al., 2014)
		Hantaan virus	*Eptesicus serotinus, Rhinolophus ferrumequinum*	(Calisher et al., 2006)
		Makokou virus	*Hipposideros ruber*	(Chen et al., 2014)
		Mouyassue virus	*Afronycteris nana, Neoromicia capensis, Neoromicia nanus*	(Chen et al., 2014)
		Puumala virus	*Rhinolophus ferrumequinum*	(Chen et al., 2014)
		Robina virus	*Pteropus alecto*	(Chen et al., 2014)
		Seoul orthohantavirus	*Hipposideros armiger, Hipposideros larvatus, Hipposideros pomona, Rhinolophus pusillus*	(Chen et al., 2014)
	Unclassified	Dode virus	*Hipposideros pomona*	(Chen et al., 2014)
		Huangpi virus	*Pipistrellus abramus*	(Chen et al., 2014)
		Magboi virus	*Nycteris hispida*	(Beltz, 2017)
		Sarawak mobatvirus	*Murina aenea*	(Chen et al., 2014)
Hepeviridae	Unclassified	Bat hepevirus	*Desmodus rotundus, Eptesicus japonensis, Eptesicus serotinus, Hipposideros abae, Hipposideros cf. ruber, Myotis bechsteinii, Myotis daubentonii, Myotis davidii, Myotis emarginatus, Mystacina tuberculata, Pipistrellus nathusii, Plecotus sacrimontis, Rhinolophus ferrumequinum, Vampyrodes caraccioli*	(Beltz, 2017; Chen et al., 2014)
Nairoviridae	*Orthonairovirus*	Ahun nairovirus	*Pipistrellus pipistrellus, Myotis mystacinus*	(Fagre and Kading, 2019)
		Bat nairovirus	*Myotis mystacinus*	(Chen et al., 2014)
		Crimean-Congo hemorrhagic fever virus	*Coleura afra, Eidolon helvum, Epomops franqueti, Hipposideros cf. caffer, Hipposideros gigas, Hypsignathus monstrosus, Micropteropus pusillus, Miniopterus inflatus, Myonycteris torquata, Myotis blythii, Myotis dasycneme, Myotis daubentonii, Nyctalus noctula, Rousettus aegyptiacus*	(Fagre and Kading, 2019)
		Gossas virus	*Tadarida* spp*.*	(Fagre and Kading, 2019)
		Issyk-Kul virus	*Chaerephon plicatus, Cynopterus brachyotis, Eonycteris spelaea, Eptesicus nilssoni, Eptesicus serotinus, Hipposideros diadema, Myotis blythii, Nyctalus noctule, Pipistrellus pipistrellus, Rhinolophus ferrumequinum, Rhinolophus horsfeldi, Rhinolophus lepidus, Scotophilus kuhlii, Taphozous melanopogon, Vespertilio serotinus, Argasid ticks collected from Vespertilio pipstrellus*	(Calisher et al., 2006; Fagre and Kading, 2019; Chen et al., 2014)
		Kasokero virus	*Rousettus aegyptiacus*	(Calisher et al., 2006; Chen et al., 2014)
		Keterah virus	*Tick larvae (Argus pusillus) collected from Scotophilus temminckii, Scotophilus kuhlii*	(Beltz, 2017; Fagre and Kading, 2019)
		Leopards hill virus	*Hipposideros gigas*	(Fagre and Kading, 2019; Chen et al., 2014)
		Uzun Agach virus	*Myotis blythii*	(Fagre and Kading, 2019; Chen et al., 2014)
		Yogue virus	*Rousettus aegyptiacus*	(Calisher et al., 2006; Chen et al., 2014)
Orthomyxoviridae	*Alphainfluenzavirus*	H3N2	*Nyctalus noctule*	(Beltz, 2017)
		H17N10	*Sturnira lilium*	(Beltz, 2017; Chen et al., 2014)
		H18N11	*Artibeus lituratus, Artibeus obscurus, Artibeus planirostris, Carollia brevicauda, Carollia perspicillata, Desmodus rotundus, Molossus molossus, Phyllostomus discolor, Phyllostomus hastatus, Platyrrhinus recifinus, Rhinophylla pumilio, Vampyressa bidens*	(Beltz, 2017; Chen et al., 2014)
Paramyxoviridae	*Henipavirus*	Cedar virus	*Pteropus poliocephalus*	(Beltz, 2017)
		Henipavirus spp.	*Acerodon celebensis, Eonycteris spelaea, Hypsignathus monstrosus, Myonycteris torquata, Pteronotus parnellii, Rousettus aegyptiacus, Rousettus madagascariensis*	(Beltz, 2017; Chen et al., 2014)
		Henipa-like virus	*Eidolon helvum*	(Beltz, 2017)
		Hendra virus	*Dobsonia andersoni, Dobsonia magna, Dobsonia moluccensis, Pteropus admiralitatum, Pteropus alecto, Pteropus capistratus, Pteropus conspicillatus, Pteropus hypomelanus, Pteropus neohibernicus, Pteropus poliocephalus, Pteropus scapulatus*	(Calisher et al., 2006; Beltz, 2017; Chen et al., 2014)
		Nipah virus	*Cynopterus brachyotis, Cynopterus sphinx, Eidolon dupreanum, Eonycteris spelaea, Epomophorus gambianus, Hipposideros larvatus, Pteropus giganteus, Pteropus hypomelanus, Pteropus lylei, Pteropus rufus, Pteropus vampyrus, Rousettus amplexicaudatus, Scotophilus kuhlii*	(Calisher et al., 2006; Beltz, 2017; Chen et al., 2014)
		Nipah-like virus	*Hipposideros pomona, Miniopterus* spp*., Myotis daubentonii, Myotis ricketti, Rhinolophus affinis, Rhinolophus sinicus, Rousettus leschenaultii*	(Beltz, 2017)
	*Pararubulavirus*	Achimota virus	*Eidolon helvum*	(Markotter et al., 2020; Beltz, 2017; Chen et al., 2014)
		Hervey virus	*Pteropus* spp*.*	(Beltz, 2017; Chen et al., 2014)
		Menangle virus	*Pteropus alecto, Pteropus conspicillatus, Pteropus poliocephalus*	(Calisher et al., 2006; Beltz, 2017; Chen et al., 2014)
		Sosuga virus	*Rousettus aegyptiacus*	(Beltz, 2017; Markotter et al., 2020; Chen et al., 2014)
		Teviot virus	*Pteropus* spp*.*	(Beltz, 2017; Chen et al., 2014)
		Tioman virus	*Pteropus giganteus, Pteropus hypomelanus, Pteropus rufus, Rousettus madagascariensis*	(Calisher et al., 2006; Beltz, 2017; Chen et al., 2014)
		Tuhoko virus	*Rousettus leschenaultii*	(Beltz, 2017; Chen et al., 2014)
	*Orthorubulavirus*	Alston virus	*Pteropus* spp*., Scotorepens balstoni*	(Chen et al., 2014)
		Mapuera virus	*Sturnira lilium*	(Calisher et al., 2006; Chen et al., 2014)
		Porcine rubulavirus	*Artibeus jamaicensis, Desmodus rotundus, Pteronotus parnellii*	(Chen et al., 2014)
	Unclassified	Bat paramyxovirus spp.	*Acerodon celebensis, Afronycteris nana, Artibeus planirostris, Cardioderma cor, Carollia brevicauda, Carollia perspicillata, Chaerephon leucogaster, Chalinolobus gouldii, Chalinolobus morio, Coleura afra, Coleura kibomalandy, Desmodus rotundus, Eidolon helvum, Epesicus hottentotus, Eptesicus regulus, Eoncyteris spelaea, Epomophorus gambianus, Glossophaga soricina, Hipposideros armiger, Hipposideros caffer, Hipposideros cineraceus, Hipposideros fuliginosus, Hipposideros gigas, Hipposideros ruber, Hypsignathus monstrosus, Kerivoula argentata, Megaloglossus woermanni, Miniopterus cf. ambohitrensis, Miniopterus fuliginosus, Miniopterus gleni, Miniopterus griveaudi, Miniopterus inflatus, Miniopterus mahafaliensis, Miniopterus mahafaliensis, Miniopterus minor, Miniopterus natalensis, Miniopterus schreibersii, Miniopterus sororculus, Mops leucostigma, Mops midas, Mormopterus acetabulosus, Mormopterus francoismoutoui, Mormopterus jugularis, Murina leucogaster, Myonycteris torquata, Myotis alcathoe, Myotis bechsteinii, Myotis blythii, Myotis capaccinii, Myotis daubentonii, Myotis emarginatus, Myotis goudoti, Myotis griveaudi, Myotis macrodactylus, Myotis mystacinus, Myotis nattereri, Myotis petax, Neoromicia nanus, Nyctalus noctule, Nycteris thebaica, Otomops madagascariensis, Otomops martiensseni, Phyllostomus hastatus, Pipistrellus kuhlii, Pipistrellus pipistrellus, Pteronotus alitonus, Pteronotus parnellii, Pteropus poliocephalus, Pteropus rufus, Pteropus vampyrus, Rhinolophus denti, Rhinolophus ferrumequinum, Rhinolophus hipposideros, Rhinolophus landeri, Rhinolophus simulator, Rhinolophus smithersi, Rhinopoma hardwickii, Rousettus aegyptiacus, Scotorepens balstoni, Scotophilus kuhlii, Sturnira lilium, Tadarida brasiliensis, Taphozous melanopogon Taphozous mauritianus, Taphozous theobaldi, Triaenops afer, Triaenops furculus, Triaenops menamena, Triaenops menamena, Triaenops menamena, Vespertilio sinensis*	(Chen et al., 2014)
		Bat parainfluenza virus	*Rousettus leschenaultii*	(Beltz, 2017)
		Belinga bat virus	*Coleura afra*	(Beltz, 2017)
		Boe paramyxovirus	*Desmodus rotundus*	(Chen et al., 2014)
		Geelong paramyxovirus	*Pteropus poliocephalus*	(Beltz, 2017; Chen et al., 2014)
		Grove virus	*Pteropus* spp*.*	(Beltz, 2017; Chen et al., 2014)
		Guato paramyxovirus	*Carollia perspicillata*	(Chen et al., 2014)
		Jeilongvirus spp.	*Hipposideros armiger, Hipposideros cineraceus, Myotis mystacinus, Pipistrellus pipistrellus, Taphozous melanopogon*	(Beltz, 2017)
		Kanhgag paramyxovirus	*Desmodus rotundus*	(Chen et al., 2014)
		Morbillivirus spp.	*Carollia brevicauda, Carollia perspicillata, Coleura afra, Glossophaga soricina, Hipposideros abae, Hipposideros caffer, Hipposideros cf ruber, Hipposideros gigas, Myotis alcathoe, Myotis bechsteinii, Myotis capaccinii, Myotis daubentonii, Myotis myotis, Myotis mystacinus, Pipistrellus cf nanus, Pteronotus parnellii*	(Beltz, 2017)
		Morbillivirus-related virus	*Chaerephon leucogaster, Coleura kibomalandy, Eptesicus hottentotus, Hipposideros fuliginosus, Kerivoula argentata, Miniopterus cf. ambohitrensis, Miniopterus gleni, Miniopterus griveaudi, Miniopterus mahafaliensis, Miniopterus minor, Miniopterus sororculus, Mops leucostigma, Mops midas, Mormopterus acetabulosus, Mormopterus jugularis, Myotis goudoti, Neoromicia nanus, Nycteris thebaica, Otomops madagascariensis, Otomops martoensseni, Paratriaenops furculus, Pipistrellus hesperidus, Pteropus rufus, Rhinolophus denti, Rhinolophus landeri, Taphozous* spp*., Triaenops afer, Triaenops menamena*	(Beltz, 2017)
		Rubulavirus spp.	*Carollia perspicillata, Desmodus rotundus, Eidolon helvum, Epomophorus minimus, Hipposideros cf ruber, Hipposideros gigas, Hypsignathus monstrosus, Megaloglossus woermanni, Miniopterus inflatus, Rousettus aegyptiacus, Rousettus leschenaultii*	(Beltz, 2017; Chen et al., 2014)
		Yarra Bend paramyxovirus	*Pteropus poliocephalus*	(Beltz, 2017)
		Yeppoon virus	*Pteropus* spp*.*	(Beltz, 2017)
Peribunyaviridae	*Orthobunyavirus*	Bunyamwera virus	*Eidolon helvum, Mops condylurus, Myotis lucifugus, Rousettus aegyptiacus*	(Fagre and Kading, 2019)
		Bimiti virus	*Anoura geoffroyi, Carollia perspicillata, Natalus tumidirostris, Phyllostomus hastatus, Pteronotus parnellii*	(Beltz, 2017)
		California encephalitis virus	*Myotis keenii*	(Fagre and Kading, 2019)
		Caraparu virus	*Artibeus lituratus*	(Beltz, 2017)
		Catu virus	*Anoura geoffroyi, Carollia perspicillata, Molossus obscurus, Phyllostomus hastatus*	(Calisher et al., 2006; Beltz, 2017; Fagre and Kading, 2019)
		Guama virus	*Anoura geoffroyi, Artibeus lituratus Phyllostomus hastatus,*	(Beltz, 2017; Fagre and Kading, 2019)
		Kaeng Khoi virus	*Chaerephon plicata, Taphozous theobaldi*	(Calisher et al., 2006; Beltz, 2017)
		Manzanilla virus	*Molossus ater*	(Beltz, 2017)
		Mojui dos Campos virus	*Unidentified bat*	(Calisher et al., 2006; Fagre and Kading, 2019)
		Nepuyo virus	*Artibeus jamaicensis, Artibeus lituratus, Phyllostomus hastaus*	(Beltz, 2017)
		Oriboca virus	*Artibeus lituratus*	(Beltz, 2017)
		Restan virus	*Artibius jamaicensis, Artibius lituratus, Carollia perspicillata*	(Beltz, 2017)
	Unclassified	Bat bunyavirus	*Molossus molossus, Rhinolophus ferrumequinum, Rhinolophus pearsoni*	(Chen et al., 2014)
		Bangui virus	*Scotophilus* spp*., Pipistrellus* spp*., Tadarida* spp*.*	(Calisher et al., 2006; Fagre and Kading, 2019)
Picobirnaviridae	Unclassified	Limbe partiti-like virus	*Eidolon helvum*	(Chen et al., 2014)
		Lysoka partiti-like virus	*Eidolon helvum*	(Chen et al., 2014)
		Moyuka partiti-like virus	*Eidolon helvum*	(Chen et al., 2014)
Picornaviridae	*Crohivirus*	Bat crohivirus	*Eidolon helvum*	(Chen et al., 2014)
	*Hepatovirus*	Hepatitis A virus	*Rhinopoma hardwickii*	(Chen et al., 2014)
		Bat hepatovirus	*Coleura afra, Eidolon helvum, Glauconcyteris* spp*., Miniopterus manavi, Miniopterus schreibersii, Myotis dasycneme, Myotis myotis, Natalus lanatus, Nyctalus noctula, Pipistrellus kuhlii, Rhinolophus ferrumequinum, Rhinolophus hipposideros, Rhinolophus landeri*	(Chen et al., 2014)
	*Kunsagivirus*	Bat kunsagivirus	*Eidolon helvum*	(Chen et al., 2014)
	*Mischivirus*	Mischivirus spp.	*Miniopterus schreibersii*	(Beltz, 2017)
	Unclassified	African bat Icavirus	*Hipposideros gigas*	(Chen et al., 2014)
		Bat picornavirus	*Desmodus rotundus, Hipposideros armiger, Miniopterus fuliginosus, Miniopterus magnate, Miniopterus pusillus, Miniopterus schreibersii, Myotis altarium, Myotis dasycneme, Myotis daubentonii, Myotis myotis, Myotis oxygnathus, Myotis ricketti, Nyctalus noctula, Nyctalus velutinus, Pipistrellus abramus, Pipistrellus pipistrellus, Rhinolophus hipposideros, Rhinolophus affinis, Rhinolophus blasii, Rhinolophus euryale, Rhinolophus ferrumequinum, Rhinolophus lepidus, Rhinolophus sinicus*	(Beltz, 2017; Chen et al., 2014)
		Bat sapelovirus	*Eidolon helvum, Myotis ricketti*	(Chen et al., 2014)
		Hubei picorna-like virus	*Unidentified bat*	(Chen et al., 2014)
		Ia Io virus	*Ia io*	(Beltz, 2017)
		Juruaca virus	*Unidentified bat*	(Calisher et al., 2006)
		Kobuvirus spp.	*Eidolon helvum, Murina ussuriensis, Scotophilus kuhlii*	(Chen et al., 2014)
		Parechovirus spp.	*Pipistrellus pipistrellus*	(Chen et al., 2014)
		Picorna-like virus spp.	*Eptesicus fuscus*	(Chen et al., 2014)
		Teschovirus spp.	*Eidolon helvum*	(Chen et al., 2014)
		Washington bat picornavirus	*Unidentified bat*	(Chen et al., 2014)
Phenuiviridae	*Phlebovirus*	Malsoor virus	*Rousettus leschenaultii*	(Fagre and Kading, 2019)
		Rift Valley fever virus	*Epomophorus labiatus, Epomops franqueti, Eptesicus capensis, Glauconycteris argentata, Hipposideros abae, Hipposideros caffer, Micropteropus pusillus, Miniopterus schreibersii, Rousettus aegyptiacus*	(Calisher et al., 2006; Fagre and Kading, 2019)
		Toscana virus	*Pipistrellus kuhlii*	(Calisher et al., 2006)
Reoviridae	*Coltivirus*	Taï Forest virus	*Chaerephon aloysiisabaudiae*	(Fagre and Kading, 2019)
	*Orbivirus*	Bukakata virus	*Rousettus aegyptiacus*	(Fagre and Kading, 2019)
		Elsey virus	*Pteropus* spp*.*	(Fagre and Kading, 2019)
		Fomede virus	*Nycteris gambiensis, Nycteris nana*	(Calisher et al., 2006; Fagre and Kading, 2019)
		Hermatsu virus	*Myotis macrodactylus*	(Fagre and Kading, 2019)
		Ife virus	*Eidolon helvum*	(Fagre and Kading, 2019)
		Japanaut virus	*Syconycteris crassa*	(Calisher et al., 2006; Fagre and Kading, 2019)
		Matucare virus	*Myotis* spp*., Noctilio* spp*.*	(Fagre and Kading, 2019)
	*Orthoreovirus*	Broome virus	*Pteropus alecto, Pteropus scapulatus*	(Calisher et al., 2006; Beltz, 2017; Chen et al., 2014)
		Cangyuan virus	*Rousettus leschenaultii*	(Chen et al., 2014)
		Kasama virus	*Lissonycteris angolensis ruwenzorii*	(Chen et al., 2014)
		Mammalian orthoreovirus spp.	*Eptesicus serotinus, Hipposideros* spp*., Miniopterus schreibersii, Myotis daubentonii, Myotis emarginatus, Myotis myotis, Myotis mystacinus, Myotis nattereri, Myotis ricketti, Nyctalus noctula, Pipistrellus pipistrellus, Pipistrellus kuhlii, Pipistrellus nathusii, Plecotus auritus, Rhinolophus affinis, Rhinolophus hipposideros, Rhinolophus pusillus, Tadarida teniotis, Taphozous melanopogon, Vespertillo murinus*	(Beltz, 2017; Chen et al., 2014)
		Mammalian orthoreovirus, reassortment	*Rhinolophus hipposideros, Rhinolophus pusillus*	(Beltz, 2017)
		Nelson Bay virus	*Pteropus poliocephalus, Rousettus aegyptiacus*	(Chen et al., 2014)
		Pulau virus	*Pteropus hypomelanus*	(Calisher et al., 2006; Chen et al., 2014)
		Xi River virus	*Rousettus leschenaultii*	(Beltz, 2017)
	*Rotavirus*	Bat rotavirus	*Carollia perspicillata, Desmodus rotundus, Eidolon helvum, Hipposideros caffer, Hipposideros gigas, Myotis daubentonii, Myotis mystacinus, Pipistrellus kuhlii, Pipistrellus pipistrellus, Rhinolophus blasii, Rhinolophus euryale, Rhinolophus ferrumequinum, Rhinolophus hipposideros, Rousettus aegyptiacus, Taphozous mauritianus*	(Chen et al., 2014)
		Rotavirus A	*Aseillscus stoliczkanus, Carollia perspicillata, Eidolon helvum, Glossophaga soricina, Hipposideros gigas, Hipposideros larvatus, Hipposideros pomona, Molossus molossus, Pteropus giganteus, Rhinolophus blasii, Rhinolophus euryale, Rhinolophus hipposideros, Rhinolophus simulator, Rhinopoma hardwickii, Rousettus aegyptiacus, Rousettus leschenaultii, Scotophilus kuhlii, Taphozous melanopogon, Taphozous perforatus*	(Chen et al., 2014)
		Rotavirus H	*Myotis dasycneme*	(Chen et al., 2014)
		Rotavirus J	*Miniopterus schreibersii*	(Chen et al., 2014)
Retroviridae	*Deltaretrovirus*	Bat deltaretrovirus	*Eptesicus fuscus*	(Hause et al., 2020)
	*Gammaretrovirus*	Bat gammaretro-virus	*Eptesicus serotinus, Eonycteris spelaea, Hipposideros larvatus, Macroglossus minimus, Megaderma lyra, Myotis ricketti, Pteropus alecto, Rhinolophus affinis, Rhinolophus hipposideros, Rhinolophus megaphyllus, Rhinolophus pearsonii, Rhinolophus pusillus, Rousettus leschenaultii, Syconycteris australis, Taphozous melanopogon*	(Chen et al., 2014; Hayward et al., 2020)
	Unclassified	Bat foamy virus	*Molossus molossus, Rhinolophus affinis*	(Chen et al., 2014)
		*Eidolon helvum* lung retrovirus	*Eidolon helvum*	(Chen et al., 2014)
		*Eidolon helvum* throat retrovirus	*Eidolon helvum*	(Chen et al., 2014)
Rhabdoviridae	*Ledantevirus*	Fikirini rhabdovirus	*Hipposideros commersoni, Hipposideros vittatatus, Macronycteris commersoni,*	(Chen et al., 2014)
		Kern Canyon virus	*Myotis yumanensis*	(Calisher et al., 2006)
		Kolente virus	*Hipposideros jonesi*	(Beltz, 2017)
		Kumasi rhabdovirus	*Eidolon helvum*	(Beltz, 2017; Chen et al., 2014)
		Mount Elgon bat virus	*Rhinolophus eloquens, Rhinolophus hildebrandtii*	(Calisher et al., 2006; Beltz, 2017; Chen et al., 2014)
		Oita virus	*Rhinolophus cornutus*	(Calisher et al., 2006; Chen et al., 2014)
		Vaprio virus	*Pipistrellus kuhlii*	(Chen et al., 2014; Lelli et al., 2018)
	*Lyssavirus*	Aravan virus	*Myotis blythii*	(Calisher et al., 2006; Chen et al., 2014)
		Australian bat lyssavirus	*Pteropus alecto, Pteropus conspicillatus, Pteropus poliocephalus, Pteropus scapulatus, Saccolaimus flaviventris*	(Calisher et al., 2006; Beltz, 2017; Chen et al., 2014)
		Bokeloh bat lyssavirus	*Myotis nattereri*	(Beltz, 2017; Chen et al., 2014)
		Duvenhage virus	*Miniopterus schreibersii, Nyctalus noctula, Nycteris thebaica, Vespertilio murinus*	(Calisher et al., 2006; Beltz, 2017; Markotter et al., 2020)
		European bat lyssavirus 1	*Barbastella barastellus, Eptesicus isabellinus, Eptesicus serotinus, Hypsugo savii, Miniopterus schreibersii, Myotis blythii, Myotis dasycneme, Myotis myotis, Myotis nigricans, Nyctalus noctule, Pipistrelles kuhlii, Pipistrelles nathusii, Pipistrellus pipistrellus, Plecotus auritus, Plecotus austiacus, Rhinolophus ferrumequim, Rousettus aegyptiacus, Tadarida teniotis, Vespertilio murinus*	(Calisher et al., 2006; Beltz, 2017)
		European bat lyssavirus 2	*Miniopterus schreibersii, Myotis dasycneme, Myotis daubentonii, Myotis myotis, Myotis nattereri, Pipistrelles nathusii, Rhinolophus ferrumequinum*	(Calisher et al., 2006; Beltz, 2017)
		Gannoruwa bat lyssavirus	*Pteropus giganteus, Pteropus medius*	(Chen et al., 2014)
		Irkut virus	*Murina leucogaster*	(Calisher et al., 2006; Chen et al., 2014 )
		Khujand virus	*Myotis mystacinus*	(Calisher et al., 2006)
		Kotalahti bat lyssavirus	*Myotis brandtii*	(Chen et al., 2014)
		Lagos bat virus	*Eidolon helvum, Epomophorus gambianus, Epomophorus wahlbergi, Epomops dobsoni, Micropteropus pusillus, Nycteris gambiensis, Rousettus aegyptiacus*	(Calisher et al., 2006; Beltz, 2017; Markotter et al., 2020)
		Lleida bat lyssavirus	*Miniopterus schreibersii*	(Beltz, 2017)
		Matlapitsi bat lyssavirus	*Miniopterus natalensis*	(Chen et al., 2014)
		Rabies virus	*Numerous bat species, essentially worldwide*	(Calisher et al., 2006)
		Shimoni bat virus	*Chaerephon pumila, Eidolon helvum, Epomophorus wahlbergi, Hipposideros commersoni, Hipposideros vittatus, Macronycteris commersoni, Miniopterus* spp*., Pipistrellus* spp*., Rousettus aegyptiacus*	(Beltz, 2017; Chen et al., 2014; Markotter et al., 2020)
		Taiwan bat lyssavirus	*Pipistrellus abramus*	(Chen et al., 2014)
		West Caucasian bat virus	*Miniopterus schreibersii*	(Calisher et al., 2006)
	*Vesiculovirus*	American bat vesicuolvirus	*Eptesicus fuscus*	(Beltz, 2017; Chen et al., 2014)
		Benxi bat virus	*Rhinolophus ferrumequinum*	(Chen et al., 2014)
		Jinghong bat virus	*Rhinolophus affinis*	(Xu et al., 2018)
		Qiongzhong bat virus	*Rhinolophus sinicus, Rhinolophus affinis,*	(Luo et al., 2021)
		Vesicular Stomatitis virus	*Artibius jamaicensis, Artibeus phaeotis, Desmodus rotundus, Sturnira lilium, Vamipyrodes caraccioli*	(Beltz, 2017)
		Yinshui Bat virus	*Rhinolophus sinicus*	(Luo et al., 2021)
	Unclassified	Bat rhabdovirus	*Eptesicus fuscus, Eptesicus isabellinus, Hipposideros armiger, Hipposideros turpis, Hypsugo savii, Miniopterus schreibersii, Myotis pequinius, Plectous auritus, Rhinolophus ferrumequinum, Scotomannes kuhlii*	(Chen et al., 2014)
		Sodak rhabdovirus	*Eptesicus fuscus*	(Chen et al., 2014)
		Taiyi bat virus	*Rhinolophus sinicus*	(Luo et al., 2021; Chen et al., 2014)
Togaviridae	*Alphavirus*	Babanki virus	*Epomophorus labiatus, Rousettus aegyptiacus*	(Fagre and Kading, 2019)
		Chikungunya virus	*Artibeus jamaicensis, Artibeus lituratus, Chaerephon pumilus, Eptesicus fuscus, Hipposideros caffer, Megaderma lyra, Pteropus giganteus, Rousettus aegyptiacus, Rousettus leschenaultii, Scotophilus* spp*.*	(Calisher et al., 2006; Fagre and Kading, 2019)
		Eastern equine encephalitis virus	*Artebius jamaicensis, Artibeus intermedius, Artibeus lituratus, Carollia perspicillata, Eptesicus fuscus, Glossophaga soricina, Myotis keenii, Myotis lucifugus, Phyllostomus hastatus, Rhynchonycteris naso, Sturnira lilium, Vampyrops helleri*	(Beltz, 2017; Fagre and Kading, 2019)
		Mucambo virus	*Carollia perspicillata, Molossus ater, Phyllostomus hastatus*	(Beltz, 2017; Fagre and Kading, 2019)
		O’nyong’nyong virus	*Chaerephon pumila, Rousettus aegyptiacus*	(Fagre and Kading, 2019)
		Ross River virus	*Pteropus poliocephalus, Pteropus scapulatus*	(Fagre and Kading, 2019)
		Semliki forest virus	*Eidolon helvum, Mops condylurus, Myotis lucifugus, Rousettus aegyptiacus*	(Fagre and Kading, 2019)
		Sindbis virus	*Eidolon helvum,* Hipposideridae spp*., Mops condylurus, Myotis lucifugus,* Rhinolophidae spp*.*	(Calisher et al., 2006; Fagre and Kading, 2019)
		Tonate virus	*Trachops cirrhosus*	(Chen et al., 2014)
		Venezuelan equine encephalitis virus	*Artibeus jamaicensis, Artibeus lituratus, Artibeus phaeotis, Artibeus planirostris, Artibeus turpis, Carollia brevicauda, Carollia perspicillata, Carollia sowelli, Carollia subrufa, Desmodus rotundus, Eptesicus fuscus, Glossophaga soricina, Noctilio leporinus, Phyllostomus discolor, Pipistrellus subflavus, Plecotus townsendii, Sturnira lilium, Sturnira ludovici, Sturnira parvidens, Uroderma bilobatum*	(Calisher et al., 2006; Beltz, 2017; Fagre and Kading, 2019)
		Western equine encephalitis virus	*Artibeus jamaicensis, Eptesicus* spp*.*	(Beltz, 2017; Fagre and Kading, 2019)
	Unclassified	Ruhugu virus	*Doryrhina cyclops*	(Chen et al., 2014)

Data from serological and molecular studies have been included. For updated information, see database of bat-associated viruses (DBatVir) (Chen et al., 2014). Classification is based on the International Committee on Taxonomy of Viruses (ICTV), NCBI taxonomy database, and/or published literature.

**Table 2:** An updated list of DNA viruses detected in bats

Viral Family	Genus	Viral species	Bat Species	Refs
Adenoviridae	*Mastadenovirus*	Bat adenovirus	*Artibeus lituratus*, *Cardioderma cor*, *Chalinolobus gouldii, Chalinolobus morio,Chaerephon pumilus*, *Coleura afra*, *Desmodus rotundus,*Doryrhina cyclops, *Eidolon helvum*, *Eptesicus nilssoni, Eptesicus regulus,Epomophorus gambianus*, *Epomops franqueti*, *Eptesicus serotinus*, *Hipposideros armiger*, *Hipposideros caffer*, *Hipposideros commersoni*, *Hipposideros gigas*, *Hipposideros ruber*, *Hypsignathus monstrosus*, *Hypsugo savii*, Ia io, Macronycteris commersoni, *Megaloglossus woermanni*, *Micropteropus pusillus*, *Miniopterus minor*, *Miniopterus natalensis*, *Mops condylurus*, Myonycteris angolensis, *Myotis emarginatus*, *Myotis fimbriatus*, *Myotis horsfieldii*, *Myotis macrodactylus*, *Myotis myotis*, *Myotis pequinius*, *Myotis ricketti*, *Neoromicia capensis*, *Neoromicia tenuipinnis*, *Nyctalus lasiopterus*, *Nyctalus leisleri*, *Nyctalus noctula*, *Nycteris grandis*, *Nycteris hispida*, *Nyctophilus geoffroyi, Nyctophilus gouldi,Otomops martiensseni*, *Pipistrellus abramus*, *Pipistrellus kuhlii*, Pipistrellus musciculus, *Pipistrellus nathusii*, *Pipistrellus pipistrellus*, *Pipistrellus pygmaeus*, *Plecotus auritus, Plecotus austriacus,*Plecotus rafinesquii, *Pteropus dasymallus*, *Rhinolophus alcyone*, *Rhinolophus euryale*, *Rhinolophus fumigatus*, *Rousettus aegyptiacus*, *Scotophilus kuhlii*, *Sturnira lilium*, *Taphozous perforatus*, *Vespertilio sinensis*	(Chen et al., 2014; Beltz, 2017; Calisher et al., 2006)
		Mastadenovirus spp.	*Myotis velifer, Neocromicia capensis, Neoromicia helios, Neoromicia nanus*	(Chen et al., 2014)
		Unidentified adenovirus	*Pteropus giganteus, Scotophilus kuhlii*	(Chen et al., 2014)
Anelloviridae	Unclassified	Torque teno virus	*Tadarida brasiliensis*	(Chen et al., 2014)
Circoviridae	*Circovirus*	Bat-associated circovirus	*Desmodus rotundus, Eumops bonariensis, Molossus molossus, Myotis frater, Myotis macrodactylus, Myotis myotis, Myotis petax, Plecotus auritus, Rhinolophus hipposideros, Tadarida brasiliensis, Vespertilio murinus*	(Chen et al., 2014)
		Bat circovirus	*Eptesicus serotinus, Hipposideros armiger, Miniopterus fuliginosus, Miniopterus schreibersii, Murina leucogaster, Myotis fimbriatus, Myotis pequinius, Myotis ricketti, Pipistrellus pipistrellus, Plecotus auritus, Rhinolophus affinis, Rhinolophus ferrumequinum, Rhinolophus hipposideros, Rhinolophus luctus, Rhinolophus pusillus, Rhinolophus sinicus, Rousettus leschenaultii, Tyloncyteris pachypus, Vespertilio sinensis*	(Chen et al., 2014)
		Circovirus spp.	*Atribeus* spp*., Macrotus waterhousii, Myotis alcathoe, Myotis emarginatus, Myotis nattereri, Nyctalus noctula, Pipistrellus nathusii, Plecotus auritus, Pteronotus parnellii, Rhinolophus pusillus*	(Calisher et al., 2006; Beltz, 2017)
	*Cyclovirus*	Bat-associated cyclovirus	*Antrozous pallidus, Chalinolobus gouldii, Eptesicus regulus, Eumops bonariensis, Molossus molossus, Tadarida brasiliensis*	(Beltz, 2017; Chen et al., 2014 )
		Bat cyclovirus	*Eidolon helvum, Tadarida brasiliensis*	(Chen et al., 2014)
		Cyclovirus spp.	*Hipposideros armiger, Neoromicia* spp*.*	(Chen et al., 2014)
Genomoviridae	*Gemykibivirus*	Bat gemycircularvirus	*Hipposideros larvatus*	(Chen et al., 2014)
		Bat-associated gemycircularvirus spp.	*Eumops bonariensis, Molossus molossus*	(Chen et al., 2014)
		Bat-associated gemykibivirus spp.	*Eumops bonariensis, Molossus molossus, Tadarida brasiliensis*	(Chen et al., 2014)
Hepadnaviridae	*Orthohepadnavirus*	Bat hepatitis B virus	*Hipposideros armiger*, *Hipposideros ruber*, *Miniopterus schreibersii*, *Myotis chinensis*, *Platyrrhinus lineatus*, *Rhinolophus affinis*, Rhinolophus alycone, *Rhinolophus ferrumequinum*, *Rhinolophus luctus*, *Rhinolophus monoceros*, *Rhinolophus pearsonii*, *Rhinolophus pusillus*, *Rhinolophus sinicus*, *Uroderma bilobatum*	(Chen et al., 2014)
	Unclassified	Bat hepadnavirus	*Hipposideros larvatus*, *Hipposideros pomona*	(Chen et al., 2014)
		Bat hepatitis virus	*Hipposideros pomona*, Miniopterus fuliginosus, *Miniopterus schreibersii*, *Myotis davidii*, *Myotis fimbriatus*, *Myotis pequinius*, *Myotis ricketti*, *Rhinolophus affinis*	(Chen et al., 2014)
Herpesviridae	*Alphaherpesvirus*	Alphaherpesvirus spp.	*Eidolon helvum*, Lonchophylla thomasi, *Pteropus lylei*	(Beltz, 2017)
	*Betaherpesvirus*	Bat betaherpesvirus spp.	*Artibeus lituratus*, *Desmodus rotundus*, *Diphylla ecaudata*, *Eptesicus diminutus*, *Eptesicus isabellinus*, *Eptesicus serotinus*, *Glossophaga soricina*, *Hypsugo savii*, Miniopterus fuliginosus, *Miniopterus schreibersii*, *Hypsugo savii*, *Molossus molossus*, *Myotis alcathoe*, *Myotis blythii*, *Myotis bechsteinii*, *Myotis daubentonii*, *Myotis escalerai*, *Myotis myotis*, *Myotis mystacinus*, *Myotis nattereri*, *Myotis oxyotus*, *Nyctalus lasiopterus*, *Nyctalus noctula*, *Pipistrellus pipistrellus*, *Pipistrellus pygmaeus*, *Plecotus austriacus*, Pteronotus alitonus, *Rhinolophus ferrumequinum*, *Rhinolophus hipposideros*, Rhychonycteris naso, *Rousettus aegyptiacus*, *Saccopteryx bilineata*, *Sturnira lilium*, *Sturnira tildae*, *Tadarida brasiliensis*, *Tadarida teniotis*	(Chen et al., 2014; Beltz, 2017)
		Cytomegalovirus spp.	*Eptesicus fuscus*, *Eptesicus isabellinus*, *Eptesicus serotinus*, *Hypsugo savii*, *Miniopterus schreibersii*, *Myotis bechsteinii*, *Myotis daubentonii*, *Myotis emarginatus*, *Myotis lucifugus*, *Myotis mystacinus*, *Nyctalus lasiopterus*, *Nyctalus leisleri*, *Nyctalus noctula*, *Pipistrellus kuhlii*, *Pipistrellus pipistrellus*, *Pipistrellus pygmaeus*, *Plecotus austriacus*, *Rousettus aegyptiacus*, *Tadarida teniotis*	(Chen et al., 2014; Beltz, 2017)
	*Gammaherpesvirus*	Bat gammaherpesvirus spp.	*Anoura geoffroyi*, *Artibeus lituratus*, *Artibeus planirostris*, Carollia prespicillata, *Desmodus rotundus*, *Diaemus youngi*, *Diphylla ecaudata*, *Eptesicus furinalis*, *Eptesicus fuscus*, *Eptesicus isabellinus*, *Eptesicus serotinus*, *Miniopterus schreibersii*, *Molossus coibensis*, *Molossus molossus*, *Molossus rufus*, *Myotis capaccinii*, *Myotis daubentonii*, *Myotis emarginatus*, *Myotis myotis*, *Myotis velifer*, *Nyctalus lasiopterus*, *Nyctalus leisleri*, Pteronotus rubiginosus, *Rousettus aegyptiacus*, *Sturnira angeli*, *Tadarida brasiliensis*, *Tadarida teniotis*	(Chen et al., 2014; Beltz, 2017)
	*Rhadinovirus*	Rhadinovirus spp.	*Eptesicus serotinus*, *Miniopterus schreibersii*, *Myotis capaccinii*, *Myotis nattereri*, *Nyctalus lasiopterus*, *Nyctalus noctula*, *Pipistrellus nathusii*, *Pipistrellus pipistrellus*, *Plecotus auritus*	(Chen et al., 2014)
	*Simplexvirus*	Bat simplexvirus spp.	Eidolon dupraenum, *Eidolon helvum*, Lonchophylla thomasi, *Pteropus lylei*,	(Chen et al., 2014)
	Unclassified	Agua Preta virus	*Carollia subrufa*	(Beltz, 2017)
		Parixa virus	Lonchophylla thomasi	(Calisher et al., 2006)
		Bat herpesvirus	*Cynopterus sphinx*, Hipposideros diaderma, *Hipposideros larvatus*, *Hipposideros pomona*, *Miniopterus natalensis*, *Miniopterus schreibersii*, *Myotis ricketti*, *Pipistrellus nanulus*, *Ptenochirus jagori*, *Rhinolophus blythi*, *Rhinolophus ferrumequinum*, *Rhinolophus hipposideros*, *Rousettus aegyptiacus*, *Scotophilus kuhlii*, *Triaenops persicus*	(Chen et al., 2014; Calisher et al., 2006)
		Dzifa herpesvirus	*Triaenops afer*	(Chen et al., 2014)
Parvoviridae	*Protoparvovirus*	Megabat bufavirus	*Pteropus vampyrus*	(Chen et al., 2014)
	*Bocaparvovirus*	Bat bocavirus	*Aselliscus stoliczkanus*, *Eptesicus fuscus*, *Eidolon helvum*, Miniopterus fuliginosus, *Miniopterus schreibersii*, *Myotis myotis*, *Rhinolophus ferrumequinum*,	(Chen et al., 2014)
		Bat bocaparvovirus	*Hipposideros pomona*, *Miniopterus schreibersii*, *Rhinolophus pusillus*, *Rhinolophus sinicus*, Rousettus leshenaultii	(Chen et al., 2014)
	*Chaphamaparvovirus*	Bat chaphamaparvovirus	Unidentified bat	(Chen et al., 2014)
	*Dependoparvovirus*	Bat adeno-associated virus	*Antrozous pallidus*, *Hipposideros armiger*, *Hipposideros larvatus*, *Miniopterus schreibersii*, *Myotis daubentonii*, *Myotis ricketti*, *Rhinolophus affinis*, *Rhinolophus ferrumequinum*, *Rhinolophus macrotis*, *Rhinolophus pusillus*, *Rhinolophus sinicus*, *Scotophilus kuhlii*	(Chen et al., 2014; Beltz, 2017)
		Bat feces-associated picorna-like virus	Unidentified bat	(Chen et al., 2014)
	Unclassified	Bat parvovirus	*Atribeus jamaicensis, Artibeus lituratus, Eidolon helvum, Hipposideros pomona, Hipposideros pratti, Miniopterus fuliginosus, Miniopterus schreibersii, Myotis daubentonii, Myotis pequinius, Myotis ricketti, Nyctalus noctula, Nyctalus velutinus, Pipistrellus kuhlii, Pipistrellus nathusii, Pipistrellus pipistrellus, Plecotus auritus, Pteropus poliocephalus, Rhinolophus lepidus, Rhinolophus ferrumequinum*	(Chen et al., 2014; Beltz, 2017)
Papillomaviridae	*Dyoxipapillomavirus*	Dyoxipapillomavirus spp.	*Rhinolophus blythi*, *Scotophilus kuhlii*	(Chen et al., 2014)
	*Psipapillomavirus*	Bat papillomavirus	*Artibeus planirostris*, Artiebus liuratus, *Eidolon helvum*, *Eptesicus isabellinus*, *Eptesicus serotinus*, *Eumops bonariensis*, *Miniopterus schreibersii*, *Molossus molossus*, *Myotis ricketti*, *Mystacina tuberculata*, *Pteropus giganteus*, *Rhinolophus ferrumequinum*, *Rousettus aegyptiacus*, *Tadarida brasiliensis*, Taphozous perforates	(Chen et al., 2014)
Polyomaviridae	Unclassified	Bat polyomavirus	*Acerodon celebensis*, *Artibeus planirostris*, *Aselliscus stoliczkanus*, *Cardioderma cor*, *Carollia perspicillata*, Chaerephon spp., *Desmodus rotundus*, *Dobsonia moluccensis*, *Eidolon helvum*, *Hipposideros larvatus*, *Hipposideros pomona*, *Miniopterus africanus*, Miniopterus fuliginosus, *Miniopterus inflatus*, *Miniopterus schreibersii*, *Molossus molossus*, *Myotis daubentonii*, *Myotis davidii*, *Myotis horsfieldii*, *Myotis lucifugus*, *Myotis pequinius*, *Otomops martiensseni*, *Pipistrellus pipistrellus*, *Pteronotus davyi*, *Pteronotus parnellii*, *Pteropus vampyrus*, *Rhinolophus affinis*, *Rhinolophus blasii*, *Rhinolophus euryale*, *Rhinolophus ferrumequinum*, *Rhinolophus hildebrandtii*, *Rhinolophus hipposideros*, *Rhinolophus pearsonii*, *Rhinolophus pusillus*, *Rhinolophus simulator*, *Rhinolophus sinicus*, *Rhinolophus thomasi*, *Rousettus aegyptiacus*, *Rousettus leschenaultii*, *Scotophilus kuhlii*, *Sturnira lilium*, *Tadarida brasiliensis*, *Taphozous melanopogon*	(Chen et al., 2014; Beltz, 2017)
		Polyomavirus spp.	*Mystacina tuberculata*, *Pteropus giganteus*,	(Chen et al., 2014)
Poxviridae	*Vespertilionpoxvirus*	Eptesipox virus	*Eptesicus fuscus*	(Beltz, 2017;Chen et al., 2014 )
	*Pteropopxvirus*	Pteropox virus	*Pteropus scapulatus*	(Chen et al., 2014)
	Unclassified	Bat poxvirus	*Eidolon helvum*, Miniopterus fuliginosus	(Chen et al., 2014; Beltz, 2017)
		Hypsugopox virus	*Hypsugo savii*	(Chen et al., 2014)

Both serological and molecular evidence are included. For updated information, see database of bat-associated viruses (DBatVir) (Chen et al., 2014). Classification is based on the International Committee on Taxonomy of Viruses (ICTV), NCBI taxonomy database, and/or published literature.

#### Innate immune response

**Figure 2:**
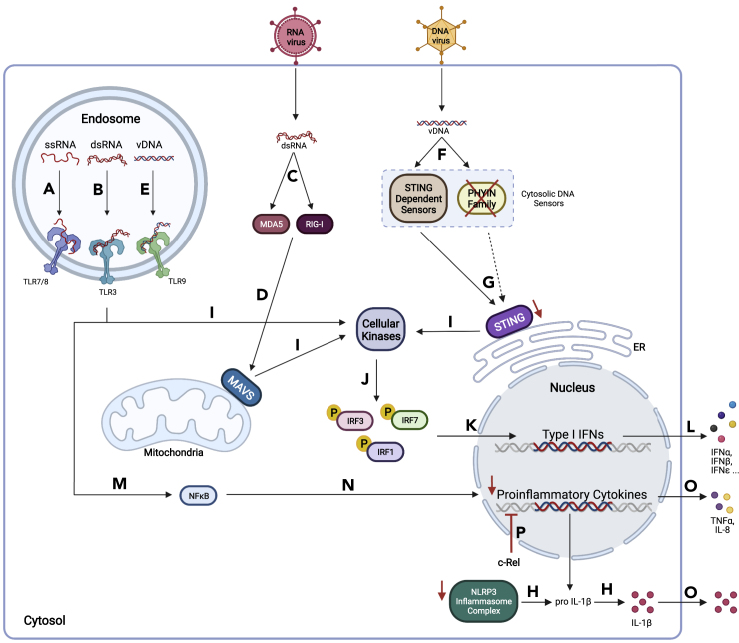
The bat innate immune response In mammalian cells, infection with single stranded (ssRNA) or double-stranded RNA (dsRNA) virus is detected by pattern recognition receptors, such as Toll-like receptors (TLRs) 3, 7, and 8 within the endosome (A, B). Studies in the black flying fox (*Pteropus alecto*) have described the existence of TLRs 3, 7, and 8 (Cowled et al., 2011); however, computational analyses for eight different bat species (*Eptesicus fuscus, Myotis brandtii, Myotis davidii, Myotis lucifugus, Pteropusalecto, Pteropus vampyrus, Rousettus leschenaultii,* and *Desmodus rotundus*) have identified unique mutations within the binding domains of TLR7 and 8 which suggest potential functional differences (Escalera-Zamudio et al., 2015). Cytoplasmic receptors, such as retinoic acid-inducible gene I (RIG-I) and melanoma differentiation-associated protein 5 (MDA5) may also detect dsRNA in the cytosol (C), leading to the activation of downstream adaptor proteins, such as mitochondrial antiviral signaling protein (MAVS) (D). Studies in *P. alecto* and *E. fuscus* cells have shown conserved structure, expression, and function for MDA5 and RIG-I (Cowled et al., 2012; Papenfuss et al., 2012; Banerjee et al., 2017). The downstream signaling cascade mediated by MAVS has not been reported for bats. Viral DNA (vDNA) present within endosomes or cytosol can be detected by TLR9 (E) and cytosolic DNA sensors (F), with the latter signaling through the stimulator of interferon genes (STING) (G). Within three different bat families (Phyllostomidae*,* Pteropodidae, and Vespertilionidae), computational analyses have discovered mutations within the binding domain of TLR9 which may alter the specificity and signaling of TLR9 in these bats (Escalera-Zamudio et al., 2015). Bats have also lost certain DNA sensors, such as the PYHIN gene family, ultimately leading to a dampened NLR-family PYRIN domain containing 3 (NLRP3)-mediated inflammasome response (Ahn et al., 2016, 2019) (H). In addition, a recent study demonstrated that bats have reduced STING activation because of a point mutation at amino acid position 358 (Xie et al., 2018), with implications for cellular responses generated against DNA virus infection and host DNA damage. Upon recognition of viral nucleic acid by TLRs, RIG-I, MDA5 and cytosolic DNA sensors, cellular kinases within the infected cell are activated (I), leading to the activation of transcription factors, like interferon (IFN) regulatory factor 1 (IRF1), IRF3 and IRF7 (J). This will ultimately lead to the induction of type I IFNs (K), such as IFNα and IFNβ, which will be secreted (L) by the infected cell to induce an antiviral state in an autocrine and paracrine manner. The existence of IRF1, 3 and 7 have been described in *P. alecto* and *E. fuscus*, and studies have demonstrated a difference in the distribution and expression pattern of IRF7 in *P. alecto* (Zhou et al., 2014, 2016a), enhanced antiviral activity of IRF3 (Banerjee et al., 2020d), and the regulation of alternate antiviral pathways by IRF1 and IRF7 (Irving et al., 2020). Signaling through TLRs may also lead to the activation of nuclear factor kappa-light-chain-enhancer of activated B cells (NF-κB) (M), which in turn induces the expression (N) and secretion of pro-inflammatory cytokines, such as tumor necrosis factor alpha (TNFα), interleukin 8 (IL-8), and IL-1 (O). While NF-κB has been described in bats, genome wide screens performed for six bat species (*Rhinolophus ferrumequinum*, *Rousettus aegyptiacus*, *Phyllostomus discolor*, *Myotis myotis*, *Pipistrellus kuhlii* and *Molossus molossus*) have demonstrated altered NF-κB signaling, which may contribute to bats having a higher tolerance toward viruses (Jebb et al., 2020). The activation of certain pro-inflammatory cytokines, such as TNFα is dampened in *E. fuscus* cells due to the presence of a c-Rel binding site in the TNFα promoter (P) (Banerjee et al., 2017). In this schematic, dampened responses are indicated by the red arrows (**↓**). This figure is not representative of a universal bat cellular response, as it has been compiled using evidence from various studies. Much remains unknown about bat cellular responses to infection and differences between bat species and with other mammalian species. ER, endoplasmic reticulum. Created with BioRender.com.

Mammals have evolved conserved pattern recognition receptors (PRRs) that sense pathogen-associated molecular patterns (PAMPs) derived from various pathogens, such as viruses (Kawai and Akira, 2006). Following the detection of a virus, a signaling cascade is initiated within the infected cell that leads to the induction of antiviral and pro-inflammatory cytokines (Figure 2). Interferons (IFNs) are antiviral cytokines that are induced by this signaling cascade. There are three groups of IFNs, designated as type I, II and III. In humans, type I and III IFNs are multigene families and are induced upon viral infection of most cell types (McNab et al., 2015; Zhou et al., 2018), while type II IFNs are secreted predominantly by natural killer cells and innate lymphoid type 1 cells and are more associated with cell-mediated immunity (Kak et al., 2018). Overall, IFNs activate the expression of downstream pro-inflammatory cytokines and interferon stimulated genes (ISGs), protecting infected and neighboring cells from further insults by invading viruses (Schoggins and Rice, 2011).

Accumulating data suggest that unlike humans, the antiviral immune response of *Pteropus alecto* relies primarily on the innate antiviral response (Beltz, 2017). *P. alecto* bats contain a contracted IFN locus compared to other mammals (Zhou et al., 2016b), suggesting an IFN response that may differ from humans. A study in *P. alecto* kidney cell lines revealed the constitutive expression of IFNα, a type I IFN, suggesting a primed antiviral state. Moreover, interferon regulatory factor 7 (IRF7), a key antiviral transcription factor, is observed to have a wider cell-type and tissue-level distribution and expression pattern in *P. alecto* compared to humans (Zhou et al., 2014, 2016a). However, this unique distribution and expression of IRF7 was not detected in other bat species, such as *Rousettus* *aegyptiacus* (Pavlovich et al., 2018)*,* although similar results have been observed in some species of fish, such as the crucian carp (*Carassius auratus L.*) (Zhang et al., 2003). Thus, different bat species may have evolved alternative antiviral strategies, including a potential shift toward an IFNω driven response relative to IFNα in at least 10 bat species (Scheben et al., 2021). Additionally, positive selection of a serine residue at the 185^th^ position in IRF3 has been shown to have enhanced antiviral properties in *E.* *fuscus* and *P. alecto* bat cells (Banerjee et al., 2020d). Some bats have also evolved alternate pathways to activate antiviral responses that are regulated by IRF1 and IRF7 (Irving et al., 2020), which may explain the inability of some coronaviruses, such as MERS-CoV, to inhibit antiviral IFN responses in *E. fuscus* bat cells (Banerjee et al., 2019).

While bat antiviral IFNs are functionally active (Crameri et al., 2009; Virtue et al., 2011; Zhou et al., 2011b, 2016a), there could be differences in the induction and level of activity of different IFN classes compared to other mammals. Recent studies have investigated the expression of IFNs and ISGs in some bat cells. Infection of *P. alecto* splenocytes with Tioman virus preferentially induced IFNλ2, a type III IFN (Zhou et al., 2011b). *P. alecto* IFNλ2 also suppressed the replication of Pulau virus (Zhou et al., 2011b). In humans, type III IFN receptors have a restricted tissue distribution pattern, where it varies widely between different organs but is restricted at the cellular level to epithelial cells (Sommereyns et al., 2008). However, type III IFN receptors in *P. alecto* have a wider distribution and expression (Zhou et al., 2011a) and both epithelial and immune cells are responsive to IFNλ treatment, suggesting a potential functional advantage of type III IFNs in *P. alecto*. The kinetics of IFN production and downstream responses may also differ in bats. For example, *P. alecto* kidney cells induce the expression of ISGs for shorter durations compared to humans, suggesting that perhaps ISGs continue to provide residual protection in *P. alecto* following the decline in gene expression. In addition, bat-specific ISGs, such as the antiviral effector 2-5A-dependent endoribonuclease (RNASEL), have been detected in both unstimulated and stimulated *P. alecto* kidney, brain, and lung cells (DeLa Cruz-Rivera et al., 2018). These findings demonstrate distinctive features of the innate immune response in some bats that may play a role in rapidly controlling high levels of virus infection. More detailed review of the bat immune response have been recently published (Banerjee et al., 2020a; Irving et al., 2021).

Infection with zoonotic bat-borne viruses, such as SARS-CoV, MERS-CoV and PEDV is associated with a dysregulated immune response and an exaggerated pro-inflammatory response in humans (for SARS-CoV and MERS-CoV) and pigs (for PEDV). Some bats have evolved multiple strategies to counteract an overt activation of the inflammatory response (Figure 2). A recent study by Ahn et al. demonstrated that *P. alecto* bats have a dampened NLR-family pyrin domain containing 3 (NLRP3), a PRR that activates inflammatory mediators during virus infection (Zhao and Zhao, 2020) and it is important in sensing Influenza A virus, Melaka virus, MERS-CoV, and SARS-CoV (Ahn et al., 2019; Chen et al., 2019). This dampened activity is noted at the level of both transcription and translation because of diminished transcriptional priming and a lowered functional capacity of all four NLRP3 isoforms (Ahn et al., 2019). In addition to NLRP3, a subsequent study analyzing the genomes of 10 bat species reported the loss of the entire PYRIN and HIN domain (PYHIN) gene family (Ahn et al., 2016). Absent in melanoma 2 (AIM2)*,* a member of the PYHIN gene family, is known to induce the activity of caspase-1, like NRLP3, leading to the cleavage of inflammatory cytokines such as interleukin-1β (Goh et al., 2020). Thus, the dampened functionality of NLRP3 and loss of the PYHIN gene family dramatically affects the activation of inflammasomes, thereby limiting the inflammatory state induced by viral infection in the described bat species.

Xie et al. have also described reduced activity in the stimulator of IFN genes (STING) (Xie et al., 2018), a PRR that plays a key role in inflammation, along with infection and cancer (Barber, 2015). This dampened activity has been described for the Chinese rufous horseshoe bat (*Rhinolophus sinicus*)*, Myotis davidii,* and *P. alecto*, and has been linked to the loss of a serine residue at amino acid position 358 in STING (Xie et al., 2018). In addition, kidney cells derived from *E. fuscus* limit expression of the pro-inflammatory cytokine, tumor necrosis factor alpha (TNFα) in response to surrogate virus infection [poly(I:C) stimulation] because of the activity of a TNFα promoter repressor protein, c-Rel (Banerjee et al., 2017). Overall, accumulating data suggest that dampened pro-inflammatory responses and extended lifespan might be the result of the co-evolution of interconnected processes and gene regulatory networks in bat species (Youm et al., 2013; Marín-Aguilar et al., 2020). It remains unknown if these evolutionary adaptations are convergent or divergent in nature.

#### Adaptive immune response

While the adaptive immune response is well characterized for humans and rodents, the lack of reagents has made it extremely challenging to study the bat adaptative immune system. However, recent developments have facilitated some advancement in this area. A first line of the adaptive immune response against invading viruses is mediated through antibodies. The major subclasses of antibodies, such as immunoglobulin M (IgM), IgG, IgA, and IgE have been detected in *P. alecto* and the large flying fox (*Pteropus vampyrus*) (Baker et al., 2010). Studies have demonstrated that antibodies against Marburg, Ebola, and Sosuga virus in convalescent sera obtained from experimentally primed *R.aegyptiacus* are non-neutralizing (Schuh et al., 2018). Similar findings were obtained upon infection of *P. alecto* with Hendra virus, where limited amounts of virus neutralizing antibodies were induced (Halpin et al., 2011). Data from limited studies suggest that bats may utilize antibodies in a manner distinct from humans to control viral infection or perhaps do not heavily rely on neutralizing antibodies to counteract invading viruses.

In addition to antibodies, cell-mediated immunity also plays an important role in the adaptive immune response in humans. Indian flying foxes (*Pteropus medius*, previously *Pteropus giganteus*) have similar lymphoid development as other mammals (Chakravarty and Sarkar, 1994). However, *P. alecto* bats have more T cells than B cells in the blood and spleen compared to humans (Periasamy et al., 2019). Moreover, *P. alecto* major histocompatibility complexes (MHC) differ from other mammals as they are predicted to accommodate larger peptides and have consensus-binding motifs that have a C-terminal proline bias (Wynne et al., 2016). This bias has been observed to aid in the detection of Hendra virus peptides (Wynne et al., 2016). In addition, the peptide binding domain in the MHC class I protein in *P. alecto* could potentially recognize numerous peptides and expresses three sequential amino acids that are absent in other mammals (methionine, aspartic acid, and leucine) (Qu et al., 2019), enhancing the activation of the cell-mediated immune response. These unique characteristics of the immune system of bats may have arisen overtime because of the evolutionary arms race between bats and viruses; however, a lot remains speculative and requires validation using mechanistic laboratory studies in more than a handful of bat species.

### Experimental *in vivo* infection studies in bats

In addition to elucidating the features of the bat immune response within structural cell types, there is a need to evaluate viral infection and antiviral responses within the host itself as it has proven to be particularly challenging to culture and study bat immune cell populations (Banerjee et al., 2018a). Though evaluating the response of wild-caught bats would best illustrate the events that occur naturally upon viral infection, establishing a wild-caught colony is not only challenging, but the increased genetic diversity adds an additional level of complexity. Therefore, hybrid models of wild-caught and laboratory bred bat colonies have been established. Nevertheless, establishing bat colonies comes with its challenges, including the requirement of a free flight enclosure, stimulation of foraging behavior, and facilitation of social characteristics of a colony (Cogswell-Hawkinson et al., 2012). In addition, bats do not produce frequent and large number of offspring (Barclay et al., 2003), limiting the number of laboratory bred bats that are available for timely experimental studies. Despite these limitations, several *in vivo* studies have been performed, and these studies demonstrate that bats do not exhibit signs of clinical disease when infected with Ebola and Marburg filoviruses, Nipah and Hendra paramyxoviruses, Dengue and West Nile flaviviruses, and coronaviruses such as MERS-CoV and SARS-CoV-2 (Paweska et al., 2016; Jones et al., 2019; Middleton et al., 2007; Woon et al., 2020; Munster et al., 2016; Davis et al., 2005; Cabrera-Romo et al., 2014; Hall et al., 2021).

Early *in vivo* studies in the 1960s evaluated the effect of temperature on Japanese B encephalitis and St. Louis encephalitis arbovirus infection in *Tadarida mexicana*, *M. lucifugus*, and *E.fuscus* bats. These studies identified that arboviruses can multiply and persist in the tissues of bats experiencing a body temperature ranging from 5 to 37°C (Sulkin et al., 1963, 1964, 1966), demonstrating a potential role of hibernating bats as a midwinter feeding source of infected blood for mosquitoes. Interestingly, transmission of Japanese encephalitis virus to *Culex* spp. mosquitoes was also noted when mosquitoes fed on infected *P. alecto* bats, although infection occurred without detectable viremia in these bats (van den Hurk et al., 2009). Because *P. alecto* exist in large colonies (Markus and Hall, 2004), it may provide enough hosts to infect local mosquitoes and aid in the maintenance of Japanese encephalitis virus (JEV). Further work is required to dissect the role of bats in the sylvatic cycle of JEV.

Experimental infection of pteropid bats has also been performed with paramyxoviruses, such as Nipah, Hendra, and Sosuga virus. During Nipah virus infection, infected grey-headed fruit bats (*Pteropus poliocephalus*) excreted low levels of virus in urine, which may sustain transmission amongst bat species where there is regular urine contamination of the fur, mutual grooming, and where urine droplets are present within the environment (Middleton et al., 2007). Meanwhile, inflammatory and degenerative changes in *P. alecto* bats inoculated with Hendra virus were limiting compared to that of a more susceptible animal model, such as the domestic ferret (*Mustela furo*) (Woon et al., 2020; Leon et al., 2018). The difference in the expression of immune-related mRNA transcripts during Hendra virus infection in *P. alecto* was also assessed, where both type I and III IFN gene expression was potentially suppressed, while *IFNα* and *IFNβ* transcripts increased significantly in the spleen of ferrets (Woon et al., 2020). The increase in type I IFNs may contribute to the inflammatory changes observed in ferrets. This study additionally supports *in vitro* findings that suggest pteropid bats have a constitutively active IFN response (Zhou et al., 2016b), as significantly higher IFNα and IFNλ levels in uninfected *P. alecto* bats compared to uninfected ferrets were observed (Woon et al., 2020). Moreover, the spleen and lungs of infected *P. alecto* bats were found to significantly express *CXCL10* transcripts, a potent chemoattractant that is believed to work in concert with type I and III IFNs (Liu et al., 2011; Woon et al., 2020). Intriguingly, this is at odds with the outcome of infection in human patients, where induction of CXCL10 was identified in patients that had succumbed to Nipah virus infection (Mathieu et al., 2012). More work is required to fully identify the kinetics and molecular determinants of henipavirus-bat and henipavirus-human interactions.

Similarly, infection of *R. aegyptiacus* with Sosuga virus did not lead to significant clinical illness; however, virus was detected in numerous tissues from two to thirteen days following inoculation (Amman et al., 2020). The highest viral load was observed in the small intestine, kidneys, colon, and rectum, suggesting that these organ systems may be a potential route of virus shedding and that contact with excretory membranes may aid in transmission from bats to humans and other animals. Moreover, evidence of infection within immunoprivileged sites, such as the eye, was noted. Recently, the eye has been associated with viral persistence in humans infected with ebolavirus, but the mechanisms that facilitate viral persistence remain unknown (Varkey et al., 2015). *R. aegyptiacus* bats have also been inoculated with avian- and bat-derived H9N2 influenza A virus (IAV) (Halwe et al., 2021). Unlike inoculation with avian-H9N2, *R. aegyptiacus* bats were susceptible to bat-H9N2, during which the first indication of infection was a change in body temperature (Halwe et al., 2021). Transmission of bat-H9N2 among experimentally infected *R. aegyptiacus* bats was evident, similarly to what has been described for *Artibeus jamaicensis* bats infected with bat-derived H18N11. However, replication of H18N11 in non-bat species, such as mice and ferrets was poor (Ciminski et al., 2019). Therefore, bats may only be susceptible to bat-borne influenza viruses and virus susceptibility, transmission, and replication could be species-specific. Despite this, the discovery of MHC-II molecules in bats and utilization of MHC-II by bat IAV subtypes H17N10 and H18N11 for cellular entry raises concerns of virus evolution and potential spillover (Karakus et al., 2019; Banerjee et al., 2020b). Although bats appear not to be susceptible to IAVs originating directly in poultry, their potential susceptibility to diverse influenza viruses should not be excluded in the absence of *in vivo* studies.

### Zoonotic spillover of viruses from bats

A series of factors are required for bat-borne viruses to successfully spillover to humans, including ecological opportunity for contact, virus-host molecular and cellular compatibility, and a permissive or circumvented immune response (Plowright et al., 2017). Although several barriers must be overcome for a successful zoonotic transfer, more than 60% of diseases in humans are caused by pathogens that originate from domestic or wild animals, with over a billion cases estimated to occur annually (Karesh et al., 2012). Some bat species have been speculated as the source of zoonotic paramyxoviruses and rhabdoviruses in humans (Johnson et al., 2015); however, additional zoonotic spillover events have been linked to bats (Table 3). Plowright et al. have recently proposed three overarching functional phases that present multiple barriers for pathogen spillover, with the probability of this event determined by disease dynamics in the reservoir host, pathogen exposure, and factors within humans that affect susceptibility to infections (Plowright et al., 2017). We have used this framework to summarize the mechanisms behind the spillover and potential spillover of known bat-borne viruses (Table 3).

**Table 3:** Bat-borne viruses with zoonotic potential

Viral Family	Genome	Virus	Diseases in humans	Transmission to humans	Refs.
Coronaviridae	ssRNA	HCoV-229E	Mild upper respiratory tract infections	Yes	(Ravelomanantsoa et al., 2020)
		HCoV-NL63	Mild upper respiratory tract infections	Yes	(Ravelomanantsoa et al., 2020)
		SARS-CoV	Severe acute respiratory syndrome	Yes, via palm civets and raccoon dogs	(Drexler et al., 2014)
		SARS-CoV-2	Coronavirus disease	Yes	(Ravelomanantsoa et al., 2020)
		SARSr-CoV	Unknown	No	
		MERS-CoV	Middle eastern respiratory syndrome	Yes, via dromedary camels	(Drexler et al., 2014)
Filoviridae	ssRNA	Ebola virus	Ebola haemorrhagic fever	Yes	(Olival and Hayman, 2014)
		Marburg virus	Marburg haemorrhagic fever	Yes	(Olival and Hayman, 2014)
Paramyxoviridae	ssRNA	Hendra virus	Hendra disease (fatal respiratory disease)	Yes, via horses	(Clayton et al., 2013)
		Menangle virus	Flu-like symptoms	Yes, via pigs	(Chant et al., 1998)
		Nipah virus	Nipah disease (severe encephalitis)	Yes, via pigs	(Clayton et al., 2013)
		Sosuga virus	Severe acute febrile illness	Yes	(Albariño et al., 2014; Amman et al., 2020)
Reoviridae	dsRNA	Kampar virus	Acute respiratory disease	Yes	(Chua et al., 2008)
		Pulau virus	Acute respiratory disease	Yes	(Chua et al., 2007)
		Mammalian orthoreovirus	Enteric + respiratory infection	Unclear	(Wang et al., 2015)
		Melaka virus	Acute respiratory disease	Yes	(Chua et al., 2007)
		Xi River virus	unknown	No	(Yamanaka et al., 2014; Du et al., 2010)
Rhabdoviridae	ssRNA	Aravan virus	unknown	No	(Banyard et al., 2014)
		Australian bat lyssavirus	Acute fatal encephalitis	Yes	(Warrilow, 2005)
		Bat mumps orthorubulavirus	unknown	No	(Katoh et al., 2016)
		Bokeloh bat lyssavirus	unknown	No	(Freuling et al., 2013)
		Duvenhage virus	Acute fatal encephalitis	Yes	(van Thiel et al., 2009)
		European bat lyssavirus 1	Acute fatal encephalitis	Yes	(Kuzmin et al., 2006; Fooks et al., 2003a)
		European bat lyssavirus 2	Acute fatal encephalitis	Yes	(Fooks et al., 2003a, 2003b)
		Irkut virus	Acute fatal encephalitis	Yes	(Banyard et al., 2014)
		Kumasi virus	No signs of disease in patient	Yes	(Binger et al., 2015)
		Khujand virus	unknown	No	(Banyard et al., 2014)
		Lagos bat virus	unknown	No	(Coertse et al., 2021)
		Lleida bat virus	unknown	No	(Aréchiga Ceballos et al., 2013)
		Rabies virus	Acute fatal encephalitis	Yes	(Johnson et al., 2010)
		Shimoni bat virus	unknown	No	(Kuzmin et al., 2010)
		West Caucasian bat virus	unknown	No	(Kuzmin et al., 2008)

This is an updated table from Allocati et al. (2016). ssRNA, single-stranded RNA; dsRNA, double-stranded RNA.

#### Pathogen pressure

Factors that are directly related to the availability of a pathogen to humans, such as pathogen dynamics, survival, and dispersal outside of the reservoir comprise pathogen pressure. Pathogen dynamics is directly impacted by the distribution, density, and the prevalence of infection among the reservoir host(s). Due to increasing human population and urbanization, animals and humans are living in closer proximity than ever before. The effects of this have already been documented for rodents, where studies in Africa have demonstrated the increased risk of rodent-borne diseases as the loss of larger wildlife has released control on rodent density (Cardillo et al., 2005; Young et al., 2014).

There is a geographical overlap of numerous bat species globally. With the destructive impact of urbanization on available roosting sites, different bat species might be forced to share co-roosting habitats (Willoughby et al., 2017), leading to new inter- and intra-species interactions. This mixing of species increases the chances of pathogen spread between naive hosts within the roost. Moreover, chiropterans are adapting to the peri-urban lifestyle caused by human expansion. Within the capital of Ghana, Accra, this adaption of bats is worrying as the city is now home to over a million African straw-colored fruit bats (*Eidolon helvum*) which are reservoirs of Lagos bat virus, henipaviruses, and Achimota virus (Hayman et al., 2008; Drexler et al., 2009; Baker et al., 2013). This heightened density of both humans and bats, in addition to the fact that hunting and the sale of bushmeat are important economic activities for this city (Kamins et al., 2011), could lead to the evolution and transmission of *E. helvum*-borne viruses, such as Achimota virus (Baker et al., 2013). This increased contact with humans is also hypothesized to favor the adaptability of bat-borne viruses, with an analysis of bat-borne coronaviruses demonstrating a heightened ability to introduce mutations which favor spillover into humans (Zhang et al., 2020; Zhou et al., 2020). Not all bat-borne zoonoses rely on direct contact with humans for spillover. In some cases, spillover into an intermediate host is a key factor for their emergence in the human population. For example, MERS-CoV is now prevalent in dromedary camels in countries in the Middle East and North and East Africa (Hemida et al., 2017), and Nipah and Menangle virus have adapted to pigs (Epstein et al., 2006; Chant et al., 1998). By infecting an intermediate host, re-emergence of these viruses in humans can occur, with rates of transmission linked to human density and animal contact.

#### Probability of infection

A successful spillover event is dependent on the genetic, physiological, and immunological attributes of the recipient human host. Regardless of their origin, the replication of a virus in its reservoir host or a new spillover host relies on the subversion of various host antiviral processes. Multiple interactions between viral and host proteins occur throughout the viral life cycle, including for viral entry, recruitment of host factors that are vital for viral replication, suppression of antiviral host processes, and egress from the infected cell to name a few. Proteomic studies for ebolavirus and Nipah virus have demonstrated that over 194 and 100 protein interactions, respectively, are required for these viruses to successfully replicate within humans (Batra et al., 2018; Martinez-Gil et al., 2017), demonstrating the number of barriers that need to be overcome for viruses to successfully replicate and adapt in non-bat hosts.

Cellular entry is the first step for a virus to be able to infect the host cell. Successful zoonotic viruses that have transmitted from bats use host molecules that are highly conserved amongst different species as receptors. For instance, bat-borne henipaviruses utilize ephrin-B2 and-B3 (Bonaparte et al., 2005; Negrete et al., 2006), filoviruses utilize the cholesterol transporter Niemann-Picktype C1 (NCP1) (Carette et al., 2011; Côté et al., 2011), and betacoronaviruses, such as SARS-CoV and SARS-CoV-2 utilize angiotensin-converting enzyme 2 (ACE2), and MERS-CoV utilizes dipeptidyl peptidase-4 (DPP4) (Li, 2013; Letko et al., 2018). These receptors are nearly identical between various bat species, intermediate hosts, and humans (Bossart et al., 2008; Ng et al., 2015; Li, 2013; Letko et al., 2018). In some instances, zoonotic viruses need to adapt to the variation in the receptor expressed by the spillover host. This ability to adapt has been noted for SARS-CoV (Li, 2013), SARS-CoV-2 (Rochman et al., 2021), and MERS-CoV (Letko et al., 2018), with previous studies demonstrating the adaptive evolution of the viral spike protein to interact with host receptors *in vivo* and *in vitro* (McRoy and Baric, 2008; Sheahan et al., 2008; Roberts et al., 2007). Similar adaptations have also been noted for ebolavirus, where Urbanowicz et al. identified advantageous mutations which emerged in the glycoprotein of ebolavirus during the West African outbreak and linked the identified amino acid substitutions to increased tropism for human cells (Urbanowicz et al., 2016). These examples of adaptive evolution provide additional support to monitor SARSr-CoVs and other potential zoonotic viruses that are circulating in bats, such as horseshoe bats (*Rhinolophus* spp*.*), to prevent the next zoonotic outbreak.

### Outstanding questions

Studies have suggested that reproduction and nutrition are key factors that affect Hendra virus seroprevalence in little red flying foxes (*Pteropus scapulatus*) (Plowright et al., 2008). Co-infection with the white-nose syndrome fungus, *Pseudogymnoascus destructans* is also associated with an increase in *Myotis lucifugus* coronavirus (Myl-CoV) replication in co-infected *M.* *lucifugus* bats (Davy et al., 2018). Indeed, accumulating data suggest that optimal bat health and low anthropogenic interference is key to limit stress in bat populations and perhaps even associated spillover of bat-borne viruses. Despite these recent advancements, we know very little about the molecular, physiological, and environmental factors that affect virus persistence and shedding in bats. Furthermore, little is known about adaptive evolution of viruses that persist in bat tissues. For instance, does prolonged exposure to temperatures experienced during hibernation and/or flight induce genetic alternations in persisting viruses? A recent *in vitro* study identified adaptions in MERS-CoV gene *ORF5* in *E.fuscus* cells that were persistently infected with MERS-CoV (Banerjee et al., 2020c). Additional studies are required to fully understand the molecular and physiological processes that facilitate virus persistence in bats.

Developing additional *in vitro* and *ex vivo* models, such as primary cells and immunocompetent organoids, along with parallel studies in whole animal models will enable a better and wholesome understanding of the bat-virus relationship. There is also a lack of collaborative studies among bat wildlife ecologists and laboratory scientists. Inter- and multidisciplinary studies will help us better understand the implications of ecological impacts on bat biology and virus persistence using data from the field, along with complementary placebo-controlled laboratory studies. One way to facilitate this would be to mimic ecological stressors at the molecular level using synthetic chemical surrogates on bat cells or organoids, followed by assessment of the impact of these stress molecules on cellular immunity and virus replication. Multicellular models, such as organoid cultures will also allow researchers to perform longitudinal studies to identify cell-to-cell spread of virus infection and cell-type specificity of cytokine production and virus replication. Currently, very few bat organoid cultures exist. Commercially available species-specific cytokines also remain limited.

Research on bats has been hampered by the lack of cross-reactive reagents (Banerjee et al., 2018a). There are over 1400 species of bats, and we have only begun studying virus-host interactions in a handful of bat species, such as *P.* *alecto*, *R.* *aegyptiacus*, *Rhinolophus* spp., *M.* *lucifugus* and *E.* *fuscus*, with researchers having access to few high-quality annotated bat genomes. By increasing the availability of annotated bat genomes, such as through efforts by the Bat1K Consortium, we can achieve a better understanding of the mechanisms that facilitate a well-tolerated antiviral immune response in bats (Teeling et al., 2018). These studies will also elucidate how these mechanisms may differ between diverse bat species, and the evolutionary role of antiviral responses in shaping viral diversity within different bat species.

There is a treasure trove of biological discoveries that are waiting to be uncovered once we develop the tools to mechanistically study bats and their viruses. For example, it remains unknown why bats only occasionally shed viruses. Understanding factors that lead to virus spillover from bats will enable the development of policies to prevent future zoonotic outbreaks and epidemics. In humans, aging is related to a deteriorating immune response (Montecino-Rodriguez et al., 2013). Bats have an exceptionally long lifespan relative to their body size and it is unknown if bats lose immune control over persistently infecting viruses as they grow older (Figure 1E) (Wilkinson and Adams, 2019; Foley et al., 2018). Could older bats be the source of increased virus shedding and cross-species transmission? Moreover, our understanding of why bats succumb to some viral infections, such as during infection with lyssaviruses and Tacaribe virus remains limited. Thus, while studies have demonstrated that bat-borne viruses that are pathogenic in other mammals can co-exist with their bat hosts in the absence of clinical disease, bats are not immortal. Some fungal and viral infections are capable of causing severe disease and death in some bat species (Cogswell-Hawkinson et al., 2012; Blehert et al., 2009; Negredo et al., 2011). Discovering the underlying factors that contribute to differential susceptibility of bat species to different viruses will lead to important ecological, immunological, and evolutionary discoveries.

It is also important to understand ecological and behavioral drivers of bat-virus interactions to understand and mitigate the spread of emerging and re-emerging bat-borne pathogens. Viruses that are found in bats, such as Nipah virus, continue to cause outbreaks in Bangladesh and Eastern India. However, in 2020, Nipah virus outbreaks were reported in Southern India for the first time (World Health Organization, 2021), a region that is thousands of kilometers away from regions of previous outbreaks. It remains unknown if bats spread Nipah virus to Southern India, and if so, what caused the virus or bat species to move over such vast geographical regions? To prevent the next pandemic, we need to invest in a One Health approach to holistically investigate ecological, behavioral, and molecular factors that regulate virus infection and shedding in wildlife species that are reservoirs of viruses with unknown zoonotic potential.

### Concluding remarks

It is apparent that the ecological, behavioral, and molecular traits possessed by bats have influenced their ability to tolerate viral infection, making them unique reservoir hosts. Through the adaption of flight, bats may have acquired responses which allow for accelerated immune processes and therefore infection control which is more rapid than other mammals (O'Shea et al., 2014). Moreover, long distance movements of bats expand their geographic range and exposure to pathogens, as bats may acquire and transmit viruses between encountered con- and hetero-specifics. Hibernation may further impact ecological interactions as seasonal roosting sites may be comprised of multiple bat species (Willoughby et al., 2017). With the origin of bats estimated to be over 64 million years ago, this heightened exposure to pathogens may have influenced the molecular differences identified in bats, allowing for evolutionary processes to generate a strengthened immune system. This increased interaction between various species may also promote the rapid adaptation of RNA viruses, facilitating host-switching.

Hibernating bats are also known to have an extended lifespan (Wilkinson and South, 2002), where longer-lived species have been predicted to carry a greater number of viruses (Guy et al., 2020). Because certain bat species possess several features which may explain their longevity, such as mechanisms which resist cancer, DNA and oxidative damage (Seluanov et al., 2018; Zhang et al., 2013; Conde-Pérezprina et al., 2012), these features may promote tolerance toward viral infections. The lifespan of bats may further influence viral diversity. Compared to RNA viruses, longevity is associated with the maintenance and spread of DNA viruses (Guy et al., 2020), which are thought to co-diverge with their hosts because of their extended duration of infection (Geoghegan et al., 2017). Meanwhile, mathematical models have predicted that social group size and taxonomical family are important for RNA viruses (Guy et al., 2020). Because RNA viruses cause infections which have a shorter duration than DNA viruses (Holmes, 2009), increased contact rate between bats may sustain transmission.

The continuous expansion of the human population is causing animals and humans to live in closer proximity and at higher density, thereby increasing the threat of zoonoses. Though certain countries may be at higher risk for the emergence and spread of zoonoses, the emergence of SARS-CoV-2 has made it clear that an outbreak that starts in one part of the world can quickly become global, affecting the health of both humans and animals. Overall, the adoption of a One Health approach across government organizations, in addition to a global surveillance system to monitor emerging infectious diseases will be an indispensable tool in our fight against zoonotic pathogens.
